# Asymmetric Effects Underlying Dynamic Heterogeneity in Miscible Blends of Poly(methyl methacrylate) with Poly(ethylene oxide)

**DOI:** 10.1021/acs.macromol.5c01587

**Published:** 2026-02-02

**Authors:** Shannon Zhang, Michael A. Webb

**Affiliations:** Department of Chemical and Biological Engineering, Princeton University, Princeton, New Jersey 08544, United States

## Abstract

The emergence of spatially variable local dynamics, or dynamic heterogeneity, is common in multicomponent polymer systems. Although often attributed to differences in the intrinsic dynamics of each component, the molecular origin of their coupling and its dependencies remain unclear. Here, we use molecular dynamics simulations of poly­(ethylene oxide) (PEO)/poly­(methyl methacrylate) (PMMA) blends, across the full range of compositions and multiple thermal regimes, to characterize local fluctuations and subchain relaxations for both PEO and PMMA. By constructing probability distributions of local composition and computing entropic measures, we connect nanoscale heterogeneity to differences in mobility between PEO and PMMA, extending beyond mean-field treatments. While PMMA segmental fluctuations in blends broadly align with *T*_g_-equivalent neat PMMA systems, PEO exhibits enhanced mobility correlated with increased free volume and broader, more diverse local compositions upon blending. Rouse-mode analysis, used to probe relaxation dynamics over different length scales, shows that PEO relaxation approaches neat-like behavior in PEO-rich domains, whereas PMMA relaxation accelerates uniformly across all mode numbers. Given the local mobility enhancement of PMMA by PEO, this uniform shift suggests a nanoscale facilitation process that extends PEO’s influence beyond its immediate environment. These findings link the statistics of local compositional heterogeneity to dynamic asymmetry across length scales, provide physical insight into the behavior of this archetypal blend system, and establish a framework for analyzing dynamic coupling in others.

## Graphical abstract

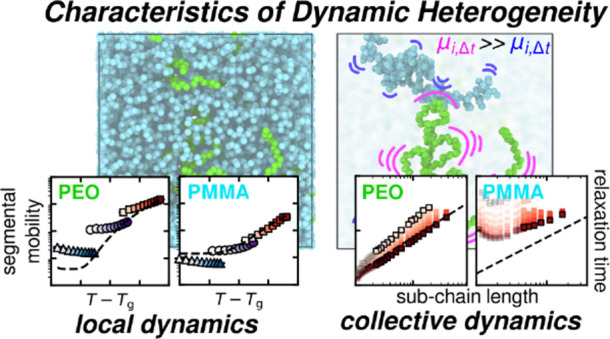

## 1 Introduction

Dynamic heterogeneity refers to spatial and temporal variations in the mobility and relaxation behavior of molecules or particles within a material. In polymer blends, such heterogeneity commonly arises due to differences in the intrinsic segmental dynamics of each component. When blended, interactions between polymers with distinct mobilities create coupled dynamical responses that manifest as perturbations across multiple spatiotemporal scales.^1–6^ Consequently, the emergence of dynamic heterogeneity can strongly influence key material properties such as the glass transition temperature (*T*_g_),^7–10^ viscoelastic behavior,^11–15^ and ion conductivity.^14,16–21^ Therefore, elucidating the molecular origins and characteristic length scales of dynamic heterogeneity is of fundamental scientific interest and may inform the design of polymer blends for targeted applications.

Dynamic heterogeneity is known to be significant in blends of poly­(ethylene oxide) (PEO) and poly­(methyl methacrylate) (PMMA). While these polymers are miscible,^22–25^ their glass transition temperatures (*T*_g_) differ by approximately 180 K.^26–28^ This dissimilarity gives rise to pronounced differences in segmental dynamics when the polymers are blended, with relaxation times differing by up to 12 orders of magnitude.^26,29^ Such contrast in mobilities has implications for applications like solid polymer electrolytes,^30–34^ where ionic conductivity is sensitive to local segmental fluctuations.^35–37^ More broadly, PEO/PMMA blends serve as model systems for investigating dynamic coupling between components at the nanoscale, with signatures of heterogeneity accessible in both simulations and experiments.^29,38–40^

Numerous experimental studies have examined the dynamics of PEO and PMMA in blends under varying conditions. Quasi-elastic neutron scattering (QENS) on blends containing up to 30 wt % PEO has shown that the segmental mobility of PMMA, on length scales up to 11 Å, is primarily governed by the temperature difference between the system and the glass transition temperature of the blend, (i.e., *T* – *T*_g_).^41–43^ In contrast, PEO dynamics are strongly influenced by interactions with PMMA, exhibiting distinct behavior at short (<1 nm) and longer (>1 nm) length scales.^38,44^ At short length scales, QENS,^39,45^ nuclear magnetic resonance (NMR),^26,29^ and neutron spin echo (NSE) spectroscopy^46^ reveal narrowing distributions of segmental relaxation times across a range of compositions and temperatures. These effects are attributed to self-concentration phenomena^45^ and local confinement by the stiffer PMMA matrix, with a dependence on local free volume.^26,29,39^

On longer length scales, PEO dynamics have been investigated using infrared dichroism and birefringence,^47^ QENS,^39,44^ and NSE.^38^ These measurements consistently indicate a pronounced slowdown of PEO segmental motion in blends with low PEO content.^38,39,44,47^ Unlike the behavior observed at shorter scales, the dynamics at these larger scales are characterized by a broad distribution of relaxation times,^39^ which is largely attributed to long-range concentration fluctuations.^47^ Collectively, these observations highlight the presence of dynamic heterogeneity across multiple length scales in PEO/PMMA blends and suggest several underlying mechanisms; however, direct inference of the molecular-level phenomena remains elusive through experimental characterization alone.

To complement experimental observations, several theoretical models have been developed to explain dynamic heterogeneity in PEO/PMMA blends. The Lodge–McLeish (LM) model, also referred to as the chain connectivity model, quantifies the influence of local self-concentration on polymer dynamics.^48^ The LM model predicts the segmental dynamics of each polymer component using the concentration within a cooperative volume centered on a monomer. The relevant length scale used to determine the size of the cooperative volume is the Kuhn length of each polymer component. It successfully predicts PEO relaxation times in blends containing 10–30 wt % PEO taken from QENS measurements for large spatial scales (*q* = 0.69 Å^–1^, approximately 18 Å) but breaks down for smaller spatial scales (*q* = 1.3 Å^–1^, approximately 10 Å).^39^ When the self-concentration is allowed to vary as a fitting parameter, the LM model reasonably fits PEO relaxation times measured by QENS^39^ and NMR,^29^ as well as terminal PMMA relaxation times.^49^ However, allowing the self-concentration to vary obfuscates the theoretical foundations of and insights from the model. Extensions of the LM model incorporate concentration fluctuations to quantitatively predict relaxation times in polymer blends.^50–53^ These studies demonstrate that concentration fluctuations are necessary to accurately capture both the peak and width of relaxation time spectra, particularly at temperatures below the blend *T*_g_. The correlation volume within which concentration fluctuations predict relaxation times is found to be on the order of the Kuhn length, supporting the assumptions of the LM model.

Another model uses mesoscale concentration fluctuations with length scales between 1 nm and 1 μm via the generalized entropy theory of glass formation, the lattice cluster theory of blend thermodynamics, and the Kirkwood–Buff theory of concentration fluctuations to predict structural relaxation times of dynamically asymmetric miscible polymers in blends.^54,55^ It can qualitatively fit the separated relaxation times of PEO and PMMA in blends but cannot account for the chemical specificity required for fitting quantitative behaviors.^54,55^

Finally, a coupling model^56–60^ and the generalized Langevin equation framework^61–63^ have been developed to describe a crossover time that separates single-chain PEO dynamics at short length scales from many-chain coupled PEO dynamics at long length scales. This framework has been used to explain unexpected phenomena and properties that arise specifically in dynamically asymmetric miscible polymer blends, such as different *T*_g_ values for each component and the breakdown of the time–temperature superposition.^58^ Additionally, the coupling model closely captures PEO segmental dynamics for experimental QENS measurements using a momentum transfer *q* value between 1 and 2 Å^–1^ in blends with 10–30 wt % PEO.^39^ These theories qualitatively capture and rationalize trends in blended PEO dynamics up to mesoscale length scales. However, like with experimental observations, they do not conclusively illustrate the molecular-level phenomena that contribute to dynamic heterogeneity.

Molecular dynamics (MD) simulations have helped to elucidate the microscopic origins of dynamic heterogeneity in polymer blends under certain conditions. Analysis of all-atom simulations with blend compositions of 10–30 wt % PEO suggest the presence of multiple populations of PEO dynamics in blends which may be caused by confinement effects of PEO in a rigid matrix of PMMA. This is deduced from van Hove self-correlation functions of hydrogen atoms from PEO, which show a double-peak structure at 400 K with an unmoving second peak^44^ and PEO relaxation times that are broadly distributed.^64^ Monitoring mean-square deviations of atoms in PEO and PMMA also suggests highly disparate local cage sizes, as the magnitude of distance traveled in the ballistic regime is much higher for PEO than PMMA.^44,64^ Additionally, PEO segmental relaxation times have been found to be more stretched upon blending than those of PMMA.^64^ Rouse analyses based on simulations of 20 wt % PEO have illustrated the non-Gaussianity of the distribution of PEO atomic displacements in blends,^65^ suggesting that local PEO segmental motion for wavelengths on the order of the size of a monomer is not strongly affected by blending but is more strongly affected at larger wavelengths.^40^ These results provide molecular-level insights into dynamic heterogeneity in PEO/PMMA blends, albeit using a varied set of compositions, temperatures, and characterization methods.

In this work, we use atomistic MD simulations to systematically characterize how dynamic heterogeneity manifests at the nanoscale in PEO/PMMA blends. A central objective is to examine how the strength of dynamic coupling between the two components depends on temperature and blend composition. Although prior studies have explored the dynamic behavior of PEO/PMMA blends, inconsistencies in system specifications (e.g., blend composition, molecular weight, and force field) and thermodynamic conditions have made it difficult to identify clear trends. To address this, we comprehensively investigate behavior over the full range of blend compositions at temperatures above, between, and below the simulated apparent *T*_g_ of both polymers. Dynamics are characterized by means of both local segmental fluctuations as well as segmental relaxation time scales. We find that blending induces asymmetric and composition-dependent changes in both local and collective dynamics, governed by temperature and local composition. In particular, the free volume and the diversity in local composition surrounding polymer units are found to correlate strongly with differences in segmental mobility between PEO and PMMA. These findings help to clarify what PEO–PMMA interactions influence dynamic heterogeneity and motivate further investigation into their generality and implications for macroscopic properties and functional performance.

## 2 Methods

### 2.1 Simulation

#### 2.1.1 General Simulation Protocols

All MD simulations are performed using LAMMPS (ver 29, Sep 2021).^66^ Systems are modeled using the all-atom optimized potentials for liquid simulations (OPLS-AA)^67^ force field. Real-space nonbonded interactions are truncated at 12 Å. Long-range electrostatics are handled using the particle-particle-particle-mesh Ewald summation method with a 10^–4^ convergence accuracy.^68,69^ Equations of motion are integrated using a velocity-Verlet integration scheme and a 1 fs time step. Periodic boundary conditions are used in all three dimensions. Temperature and pressure are controlled using a Nosé–Hoover thermostat and barostat with damping constants of 100 and 2000 fs, respectively.

#### 2.1.2 Polymer Chain Generation

Each PEO chain consists of 75 monomers, and each PMMA chain consists of 33 monomers. Here and throughout the text, the term monomer is used to refer to a single constitutional repeat unit of the polymer. As a result, chains for both polymers possess molecular weights of approximately 3300 g/mol. Syndiotactic PMMA was used in all simulations. This choice avoids the need to stochastically generate atactic sequences. Moreover, syndiotactic and atactic PMMA have been previously noted to exhibit similar *T*_g_,^70^ which may suggest similar dynamical characteristics.

Initial chain configurations are generated to approximate the expected relationship for the mean-squared end-to-end distance in that $\langle R^{2}\rangle =Ll_{k}$ , where $l_{k}$ is the Kuhn length of the polymer and *L* is the statistical contour length. In practice, rather than *L* we use *L*_ext_ ≈ ∑_*i*_ *l*_*i*_, where *L*_ext_ is the length of a fully extended polymer chain approximated by the summation of individual bond lengths *l*_*i*_. The individual *l*_*i*_ are obtained from repeat units, following geometry optimization in Avogadro 1.2.0 with the steepest descent algorithm and the UFF force field^71,72^ ; this yields *L*_ext_ values of 326.1 Å for PEO and 102.5 Å for PMMA. While this approach will initially yield somewhat more extended conformations than targeted as *L*_ext_ > *L*, this approximation is used only for initialization, and subsequent equilibration mitigates initial bias in chain dimensions. The Kuhn lengths used are 8.2 Å for PEO and 13.8 Å for PMMA.^73^ Chains are constructed by sequentially adding monomers, with each new monomer positioned based on a randomly sampled dihedral angle. For PEO, dihedral angles are drawn from a uniform distribution between −0.65 and 0.65 radians; for PMMA, the range is −0.4 to 0.4 radians. For each system described below, this process is repeated until the required number of independent chain configurations within a threshold of 5 Å of the target ⟨*R* ^2^⟩ is obtained. These configurations are used as described in Section 2.1.3. From these configurations, statistical contour lengths are also estimated by measuring the distance between two monomers and multiplying by the total number of monomers. This yields estimated contour lengths of 273 Å for PEO and 92 Å for PMMA. Dividing these values by the respective Kuhn lengths gives 33.3 and 6.7 Kuhn steps for PEO and PMMA chains, respectively. Relevant chain characteristics for PEO and PMMA are provided in Table 1 for clarity.

**Table 1:** Summary of Structural and Conformational Parameters

characteristic	PEO	PMMA
number of monomers	75	33
molecular weight (g/mol)	3319	3300
number of Kuhn steps^a^	33.3	6.7
monomers per Kuhn step^a^	2.25	4.92

a Based on Kuhn lengths from ref 73.

#### 2.1.3 System Preparation

Systems are specified in terms of their composition, given by the fraction of PEO chains relative to all chains in the simulation cell. We use the notation

$$x^{\left(\mathrm{PEO}\right)}\equiv \frac{N^{\left(\mathrm{PEO}\right)}}{N^{\left(\mathrm{PEO}\right)}+N^{\left(\mathrm{PMMA}\right)}}$$ 1

where *N* ^(PEO)^ and *N* ^(PMMA)^ are the number of chains of PEO and PMMA. Because the PEO and PMMA chains possess similar molecular weights, *x* ^(PEO)^ is also comparable to the mass fraction of PEO in the system. Condensed-phase systems are then prepared for *x* ^(PEO)^ = 0, 0.1, 0.2, 0.3, 0.4, 0.5, 0.6, 0.7, 0.8, 0.9, and 1.0. Each system contains *N* ^(PEO)^ + *N* ^(PMMA)^ = 40 total chains. For each *x* ^(PEO)^, three independent systems are prepared, resulting in a total of 11 × 3 = 33 systems.

For each system, an initial configuration is generated by randomly placing each preconstructed chain into a 60 × 60 × 60 Å simulation box with random positions and orientations. A brief energy minimization is then performed where the system is simulated in the microcanonical (NVE) ensemble with a constrained maximum distance of 0.005 Å moved per time step; this minimization takes place for 0.05 ns. After, the constrained maximum distance is increased to 0.1 Å and the system is simulated for 0.5 ns. This procedure resolves unfavorable atomic overlaps introduced by the random packing procedure. Following minimization, initial velocities are randomly generated from a uniform distribution over the interval [−0.5, 0.5], rescaled such that temperature as estimated from the kinetic energy corresponds to 300 K, and finally shifted to remove any net linear momentum in the simulation cell. The system then undergoes 1 ns of simulation in the NVE ensemble, followed by 1 ns in the canonical (NVT) ensemble at 300 K. Subsequently, a barostat is introduced to maintain the pressure at 1 atm, and the system is heated from 300 to 700 K at a rate of 80 K/ns. After reaching 700 K, the system is equilibrated in the isothermal–isobaric (NPT) ensemble for 50 ns. Finally, the system is cooled from 700 to 100 K at a rate of 10 K/ns at constant pressure.

The cooling trajectories are used for analysis of glass transition temperatures (see Section 2.2.1). Simulation configurations are also recorded specifically around the temperatures of 500, 360, and 220 K. From these configurations, additional simulations are performed for 35 ns in the NPT ensemble. These simulation trajectories are used to characterize dynamic heterogeneity by means of local segmental mobilities, as described in Sections 2.2.2, 2.2.3, and 2.2.4. Finally, additional simulation is performed for systems at 500 K to enable the Rouse mode analysis described in Section 2.2.5; for *x* ^(PEO)^ = 1.0, trajectories are extended by 100 ns, and for all other systems, trajectories are extended by 200 ns, with the difference being due to slower relaxation.

All blends are prepared well-mixed and remain so for the duration of the simulation. All systems, including neat PEO and PMMA, remain amorphous throughout our simulations. Crystallization is neither observed nor expected on the time scale of our simulations. Partially by consequence, demixing is also not observed across the full composition range. As a result, the data presented are representative of hypothetical miscible systems. The simulations do not establish miscibility for PEO/PMMA blends in this regime but rather explore the consequences of it in a simulation setting.

### 2.2 Analysis

#### 2.2.1 Glass Transition Temperature

To obtain an apparent glass transition temperature (*T*_g_), we use simulated dilatometry and analyze the temperature dependence of the specific volume, *v*(*T*), for each system. An apparent *T*_g_ is identified by a change in the slope of *v*(*T*),^74,75^ which reflects a shift in thermophysical relaxation behavior for the monitored quantity; this is associated with transition from a melt to a more glassy state. During the cooling phase of system preparation (Section 2.1.3), configurations are recorded at 10 K intervals. For each temperature, we perform an additional 1 ns simulation under NPT conditions, starting from the corresponding configuration. The average specific volume is computed from the second half of each trajectory, yielding a set of (*T*_*i*_, *v̅*_*i*_) pairs.

The apparent *T*_g_ from this data set is determined using a previously reported bootstrap resampling procedure.^20,21^ We first identify, by visual inspection, a maximum and minimum temperature within the melt regime, *T*_max_^m^ and *T*_min_^m^, and a maximum and minimum temperature within the glassy regime, *T*_max_^g^ and *T*_min_^g^. The temperatures *T*_min_^m^ and *T*_max_^g^ define sampling ranges for the melt, [*T*_min_^m^, *T*_max_^m^ = *T*_min_^m^ + 100 K], and for the glass, [*T*_min_^g^ = *T*_max_^g^ – 100 K, *T*_max_^g^]. The temperature bounds used for each blend composition are listed in the Supporting Information, Table S1. In each resampling iteration, a lower bound *T*_lo_^m^ is randomly selected from the melt range and an upper bound *T*_hi_^g^ from the glass range. Linear regressions are then performed on the data within [*T*_lo_^m^, *T*_max_^m^] and [*T*_min_^g^, *T*_hi_^g^] to fit the melt and glassy branches, respectively. The intersection of these fits is considered as one *T*_g_ sample. This process is repeated 10,000 times to generate a distribution of *T*_g_ values. We report the mean of this distribution as the estimated *T*_g_ and its standard deviation as the associated uncertainty. Simulated *v*(*T*) data and representative *T*_g_ values for each blend composition are provided in the Supporting Information, Figure S1.

#### 2.2.2 Local Segmental Mobility

To characterize local segmental dynamics, we define a segmental mobility parameter, μ_*i*,Δ*t*_, which relates to the mean-square fluctuation of the positions of a particle over a given observation time Δ*t*. Here and throughout our analysis, particles correspond to atomic centers, although analyses may be rationally extended to pseudoatoms or coarse-grained particles. For a given particle *i*, a mobility is computed as

$$\mu_{i,\Delta t}=\frac{\langle ({\vec{r}}_{i}(t)-\langle {\vec{r}}_{i}\rangle_{\Delta t}{)}^{2}\rangle_{\Delta t}}{\Delta t}$$ 2

where *i* is a backbone carbon, ${\vec{r}}_{i}(t)$ is the position of particle *i* at time *t*, and ⟨·⟩_Δ*t*_ denotes an ensemble average over the observation time. For the analysis herein, Δ*t* = 100 ps, which is substantially shorter than time scales for chain diffusion. Consequently, eq 2 mostly captures local segmental fluctuations. This quantity is computed at 500, 360, and 220 K from the final 5 ns of the 35 ns trajectories described in Section 2.1.3. Analysis based on μ_*i*,Δ*t*_ across compositions and temperatures manifests in two ways. In Section 3.2, the segmental mobility is computed at the species level by averaging over all backbone carbons of each polymer type. In Section 3.3.1, the segmental mobility is further resolved based on the local environment of each backbone carbon atom to account for compositional heterogeneity introduced by blending.

To characterize the local environment of a particle in Section 3.3.1, we define a normalized self-density parameter, ϕ̃_*i*_^(*A*)^, which measures the local enrichment of species *A* around a particle of the same species. For a given backbone carbon atom *i*, this quantity is computed as

$${\tilde{\phi }}_{i}^{\left(A\right)}=\frac{\sum_{j=1}^{n}\omega_{ij}^{\left(A\right)}\delta_{\alpha_{j}}^{\left(A\right)}}{{\left\langle \sum_{j=1}^{n}\omega_{ij}^{\left(A\right)}\right\rangle }_{x^{\left(A\right)}=1}}$$ 3

In eq 3, the numerator sums over all *n* monomers in the system, applying position-dependent weights ω_*ij*_^(*A*)^ and selecting only those monomers of species *A* via the Kronecker delta δ_α_*j*__^(*A*)^, where α_*j*_ denotes the species identity of monomer *j*. The denominator provides a normalization by the average local density around a particle in a pure *A* system (subjected to the same weighting coefficients), denoted by ⟨·⟩_*x* ^(*A*)^=1_. This normalization provides natural limits then of ϕ̃_*i*_^(*A*)^ = 1 corresponding to a local environment identical to that in a pure system of species *A*, while ϕ̃_*i*_^(*A*)^ = 0 indicates a local environment composed entirely of the other species.

To utilize eq 3, a scheme for the weighting coefficients must be defined, of which there are many reasonable choices. We choose to define a smoothing kernel of the form

$$\omega_{ij}^{\left(A\right)}=\{\begin{array}{ll} 1 & r_{ij}\leq r_{m}^{\left(A\right)}\\ \exp\left(-\frac{{\left(r_{ij}-r_{m}^{\left(A\right)}\right)}^{2}}{\sigma^{2}}\right) & r_{ij}>r_{m}^{\left(A\right)} \end{array}$$ 4

where $r_{ij}=|{\vec{r}}_{i}-{\vec{R}}_{j}|$ is the distance between particle *i* and the center of mass of monomer *j* (using the minimum image convention), and *r*_m_^(*A*)^ and σ are parameters that define a smoothing kernel. To emphasize spatially local interactions, we set *r*_m_^(*A*)^ to the radius of gyration of a single constitutional repeat unit of species *A*, computed from simulations in vacuum at room temperature. This yields *r*_m_^(PEO)^ = 1.59 Å and *r*_m_^(PMMA)^ = 2.34 Å. The smoothing width parameter is set to σ = 12 Å based on the nonbonded, real-space interaction cutoff.

#### 2.2.3 Free-Volume Analysis

The concept of free volume is often invoked to elucidate facets of polymer dynamics.^76–79^ To quantify the free volume associated with each polymer chain, we implement the following procedure. First, the simulation cell is tessellated using Delaunay triangulation, such that each simplex (tetrahedron) is defined by four atoms. Each chain is associated with a subset of simplices that have at least one vertex belonging to an atom on that chain. Next, the entire simulation cell is filled with a three-dimensional grid of *n* equally spaced spherical probes, where *n* depends on a chosen probe radius. Each probe is then classified as occupied or unoccupied based on overlap with any atom in the system, using atomic diameters defined by the σ parameters from the OPLS-AA force field. Finally, the free volume of a given chain is then computed as the total volume of unoccupied probes that fall within the chain-associated simplices. Free volume is computed and averaged over the final 5 ns of simulations equilibrated at 500, 360, and 220 K. In the main text, results correspond to a probe radius of 0.5 Å; additional results for other probe sizes are provided in Figure S2.

#### 2.2.4 Distribution of Local Composition

To further probe how packing influences polymer dynamics, we perform an analysis akin to that described by the Lodge–McLeish (LM) chain connectivity model, which assumes that a cooperative volume of spanned by a Kuhn length $l_{k}$ governs local self-concentration effects.^48^ Here, we go beyond mean-field average self-concentrations and calculate the distribution of local compositions surrounding PEO and PMMA backbone carbons within spheres of radius equal to the Kuhn length, $l_{k}$ .^80^ For PEO, we use $l_{k}$ = 8.2 Å; for PMMA, we use $l_{k}$ = 13.8 Å.^73^

The calculation proceeds similarly to that described in Section 2.2.3. First, the simulation cell is filled with *n* spherical probes distributed on a simple cubic lattice; *n* depends on the probe radius, which is 0.5 Å for our analysis. Each probe is subsequently classified as PEO, PMMA, or unoccupied based on whether the center-to-center distance between a probe and a particle is less than the atomic diameter; atomic diameters are based on the values of σ as prescribed by the employed force field; probes are also assigned to a given polymer chain based on which atom overlaps with the probe.

For each backbone carbon of polymer type *A*, we then quantify three local composition measures within the $l_{k}$ sphere: the intramolecular volume fraction ϕ_intra_^(*A*)^ (i.e., contributions from the same chain), the intermolecular PEO volume fraction ϕ_inter,PEO_^(*A*)^ (i.e., contributions from surrounding PEO chains), and the intermolecular PMMA volume fraction ϕ_inter,PMMA_^(*A*)^ (i.e., contributions from surrounding PMMA chains). These quantities are calculated as the sum of volumes of appropriately classified probes divided by the total sphere volume. The total sphere volume is defined as the sum of intramolecular, intermolecular PEO, intermolecular PMMA, and free volume contributions within the relevant sphere. This normalization is slightly different from prior analyses of a similar nature, where normalization was done on a species-specific basis.^80^ Distributions of volume fractions are computed and averaged over the final 5 ns of trajectories equilibrated at 500, 360, and 220 K. For this calculation, backbone carbon atoms are chosen simply as reference sites to ensure consistency and mitigate potential artifacts from size differences between chemical groups. However, additional calculations using alternative reference atoms yield nearly identical results (Figure S10), owing to local correlations, suggesting that derived trends are robust to such choices.

To further characterize these distributions, we calculate their Shannon entropy:

$$H(x^{\left(\mathrm{PEO}\right)};\phi_{\kappa }^{\left(A\right)})=\int P(\phi_{\kappa }^{\left(A\right)})\log_{2}[P(\phi_{\kappa }^{\left(A\right)})]d\phi_{\kappa }^{\left(A\right)}$$ 5

where *P*(ϕ_κ_^(*A*)^) is the probability distribution of local volume fraction of atoms near a backbone carbon of type *A* under condition κ (intramolecular, intermolecular PEO, or intermolecular PMMA). For interpretability, eq 5 is normalized by the Shannon entropy of a uniform distribution to yield *H̃*(·). This normalization sets *H̃* = 0 for a Dirac delta distribution and *H̃* = 1 for a uniform distribution. Thus, broader and more heterogeneous local environments drive *H̃* toward unity, while narrower or more uniform environments drive it toward zero.

#### 2.2.5 Segmental Relaxation

To investigate how dynamic heterogeneity manifests in collective polymer dynamics, we extract characteristic relaxation times associated with various subchain lengths of the polymers. In particular, we employ Rouse mode coordinates as a convenient set of collective variables that reflect different segmental length scales. For a polymer comprised of *N* monomers, Rouse mode coordinates are computed by

$${\vec{X}}_{p}(t)=\sqrt{\frac{c_{p}}{2}}\sum_{i=0}^{N-1}{\vec{r}}_{i}(t)\cos\left[\frac{p\pi }{N}\left(i+\frac{1}{2}\right)\right]$$ 6

where *p* = 0, ···, *N* – 1 indicates the mode index, ${\vec{r}}_{i}(t)$ is the position of the center of mass of the *i*th monomer on the chain at time *t*, and *c*_*p*_ is a *p*-dependent constant, such that *c*_1_ = 1 and *c*_*p*_ = 2 for all other modes.^81–85^ Importantly, in our analysis, we use the positions of chemical monomers in eq 6, rather than those of Rouse beads. By consequence, the resulting ${\vec{X}}_{p}(t)$ do not correspond to normal modes, as they do in the Rouse model theory. Based on the number of monomers present in each chain (Table 1), this yields coordinates for *p* ∈ [0, 74] for PEO and *p* ∈ [0, 32] for PMMA. The zeroth mode corresponds to the behavior of the center of mass of a chain, while all other modes roughly correspond to the collective behavior of subchains of $\frac{\left(N-1\right)}{p}$ segments.

The Rouse mode coordinates are used to compute time autocorrelation functions (ACFs), which characterize the relaxation time scales of subchains of varying segment lengths. Each ACF is fit to a stretched exponential function^40,86^ :

$$\frac{\left\langle {\vec{X}}_{p}(t)\cdot {\vec{X}}_{p}(0)\right\rangle }{\left\langle {\vec{X}}_{p}(0)\cdot {\vec{X}}_{p}(0)\right\rangle }=\exp\left[-{\left(\frac{t}{\tau_{p}}\right)}^{\beta_{p}}\right]$$ 7

where τ_*p*_ and β_*p*_ are fitting parameters. In simulations, Rouse mode autocorrelation functions for polymer chains are commonly better described by stretched exponentials than simple exponentials.^82–84,87,88^ Rather than fitting ACFs to eq 7, however, ACFs are fit to a linearized form of eq 7 for simplicity. Taking the natural logarithm yields

$$\ln\left[-\ln\left(\frac{\left\langle {\vec{X}}_{p}(t)\cdot {\vec{X}}_{p}(0)\right\rangle }{\left\langle {\vec{X}}_{p}(0)\cdot {\vec{X}}_{p}(0)\right\rangle }\right)\right]=\beta_{p}\ln(t)-\beta_{p}\ln(\tau_{p})$$ 8

allowing extraction of β_*p*_ and τ_*p*_ via linear regression. An effective relaxation time is then calculated as

$$\tau_{p}^{\mathrm{eff}}=\int_{0}^{\infty }\exp\left[-{\left(\frac{t}{\tau_{p}}\right)}^{\beta_{p}}\right]dt=\tau_{p}^{1/\beta_{p}}\Gamma \left(\frac{\beta_{p}+1}{\beta_{p}}\right)$$ 9

where Γ(·) denotes the gamma function. To perform the fitting, the ACFs were computed over time intervals of 8 ns for PEO, while for PMMA, intervals were 125, 75, and 20 ns for *x* ^(PEO)^ = 0.0, *x* ^(PEO)^ = 0.1–0.2, and *x* ^(PEO)^ = 0.9–0.3, respectively. These intervals were selected to ensure that the average normalized ACF of the largest Rouse mode for each species decayed to at least 0.2 at 500 K, to facilitate reliable fitting. Representative ACFs and fitted curves, using both simple and stretched exponentials, are shown in the Supporting Information (Figures S13, S14, S16, and S17); fits obtained with regularization are shown in Figures S19 and S20.

## 3 Results and Discussion

### 3.1 Composition-Dependent Glass Transition Temperatures

We begin by briefly comparing experimental and simulated trends regarding *T*_g_ for these PEO/PMMA systems as a function of composition. Figure 1 shows that both the simulated apparent *T*_g_ as well as the experimental *T*_g_ values^89^ follow the Fox equation

$$\frac{1}{T_{g}}=\frac{w^{\left(\mathrm{PEO}\right)}}{T_{g}^{\left(\mathrm{PEO}\right)}}+\frac{w^{\left(\mathrm{PMMA}\right)}}{T_{g}^{\left(\mathrm{PMMA}\right)}}$$ 10

where *w* ^(*i*)^ is the mass fraction of species *i* and *T*_g_^(*i*)^ is the *T*_g_ of a pure neat system of species *i*. The PMMA used in the experimental measurements of *T*_g_ are of unspecified tacticity.^89^ The apparent *T*_g_ values extracted from simulation are systematically higher than experimental values by approximately 100 K. This offset is roughly consistent across the range of examined compositions and is generally expected due to the much faster cooling rates and shorter observation times inherent to molecular simulations.^90,91^ Given this systematic disparity, the similarity in trends and alignment with the Fox equation suggests that the employed force field captures the essential physics governing blend dynamics and responds appropriately to changes in composition.

**Figure 1:**
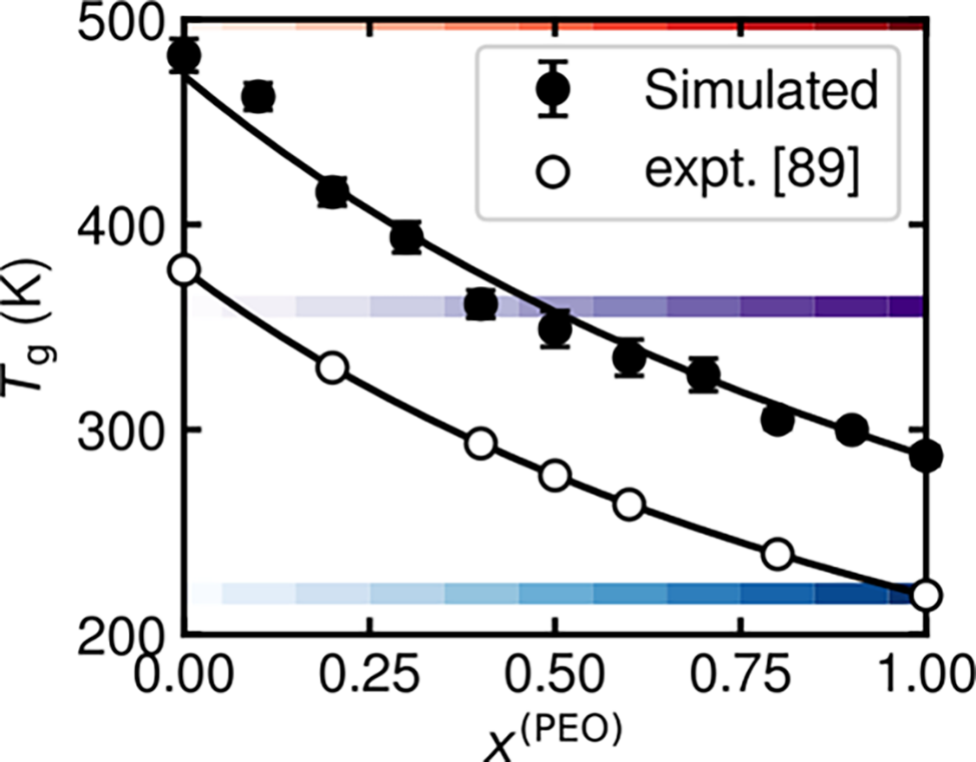
Dependence of apparent glass transition temperature (*T*_g_) on blend composition. Markers represent simulated (filled) and experimental (empty) *T*_g_ values for blends of PEO and PMMA. Experimental results are from ref 89. Solid black lines are fits to the Fox equation. Error bars reflect standard errors calculated from three independent system configurations. Horizontal, colored bands provide visual reference to 500 K (red), 360 K (purple), and 220 K (blue), which are examined in subsequent figures. The color gradation within each band distinguishes blends at the same temperature but different compositions.

Experiments using differential scanning calorimetry (DSC) on PEO/PMMA blends have reported both single^89,92,93^ and dual^27,94^ *T*_g_ signals at intermediate compositions, with the former attributed to broad, overlapping peaks arising from local composition fluctuations.^94^ In general, rapid quench rates and finite system sizes inherent to simulations may obscure multiple signatures observed experimentally. Our simulations yield a single apparent *T*_g_ based on the systematic fitting procedure described in the Supporting Information (Section S1). However, recent simulation work^28^ suggested two *T*_g_ values from density-temperature data using hyperbolic fitting. Our data (Figure S1) illustrate a broad transition window at intermediate compositions that could support hyperbolic fitting with two distinct inflection points. Regardless of whether the simulated dilatometry yields one or two apparent *T*_g_, the key outcome is that the simulations produce a reasonable composition dependence, suggesting it can capture asymmetries in PEO and PMMA dynamics.

Figure 1 also highlights three temperatures that are of specific interest in the following sections. These temperatures are selected to span distinct thermal regimes: (i) 220 K lies below the apparent *T*_g_ of both pure components and therefore below that of any blend; (ii) 360 K falls between the *T*_g_ values of pure PEO and PMMA, such that some blends are above and others below their respective *T*_g_; and (iii) 500 K exceeds the *T*_g_ of both pure components and all blends. This temperature range enables the examination of how interspecies dynamical coupling depends not only on the different *T*_g_ values of PEO and PMMA but also on the absolute temperature with respect to these *T*_g_ values.

### 3.2 Characterization of Species-Dependent Local Dynamics

As an initial characterization of nanoscale dynamic heterogeneity, we examine how blending influences the average local dynamics of PEO and PMMA compared to their behavior in neat systems. Specifically, we analyze species-resolved segmental mobilities (μ_*i*,Δ*t*_) of polymer segments across varying blend compositions at temperatures below (220 K), between (360 K), and above (500 K) the apparent *T*_g_ values of the pure components.

Figure 2A,B shows the μ_*i*,Δ*t*_ as a function of *T* – *T*_g_, where *T*_g_ varies with composition. The dynamics for PEO in blends (Figure 2A) exhibit significant deviations from neat behavior at the same distance from *T*_g_ (dashed black line) across most compositions but particularly in PMMA-rich blends (low *x* ^(PEO)^). This indicates that *T* – *T*_g_ is not a reliable predictor of local segmental dynamics for PEO. In contrast, PMMA (Figure 2B) dynamics in blends closely follow the behavior of neat PMMA, albeit with less strong correlation at temperatures below *T*_g_. This trend is consistent with previous findings suggesting that PMMA dynamics are effectively governed by the temperature difference from the *T*_g_ of the blend^41–43^ and also reveal an asymmetry in dynamical coupling between PEO and PMMA. The mobilities of both PEO and PMMA at 220 K (below *T*_g_ of both pure components) decrease with increasing *x* ^(PEO)^. This behavior is both opposite to the behavior at 360 and 500 K and also possibly unexpected because PEO is the higher-mobility species in the blend.

**Figure 2:**
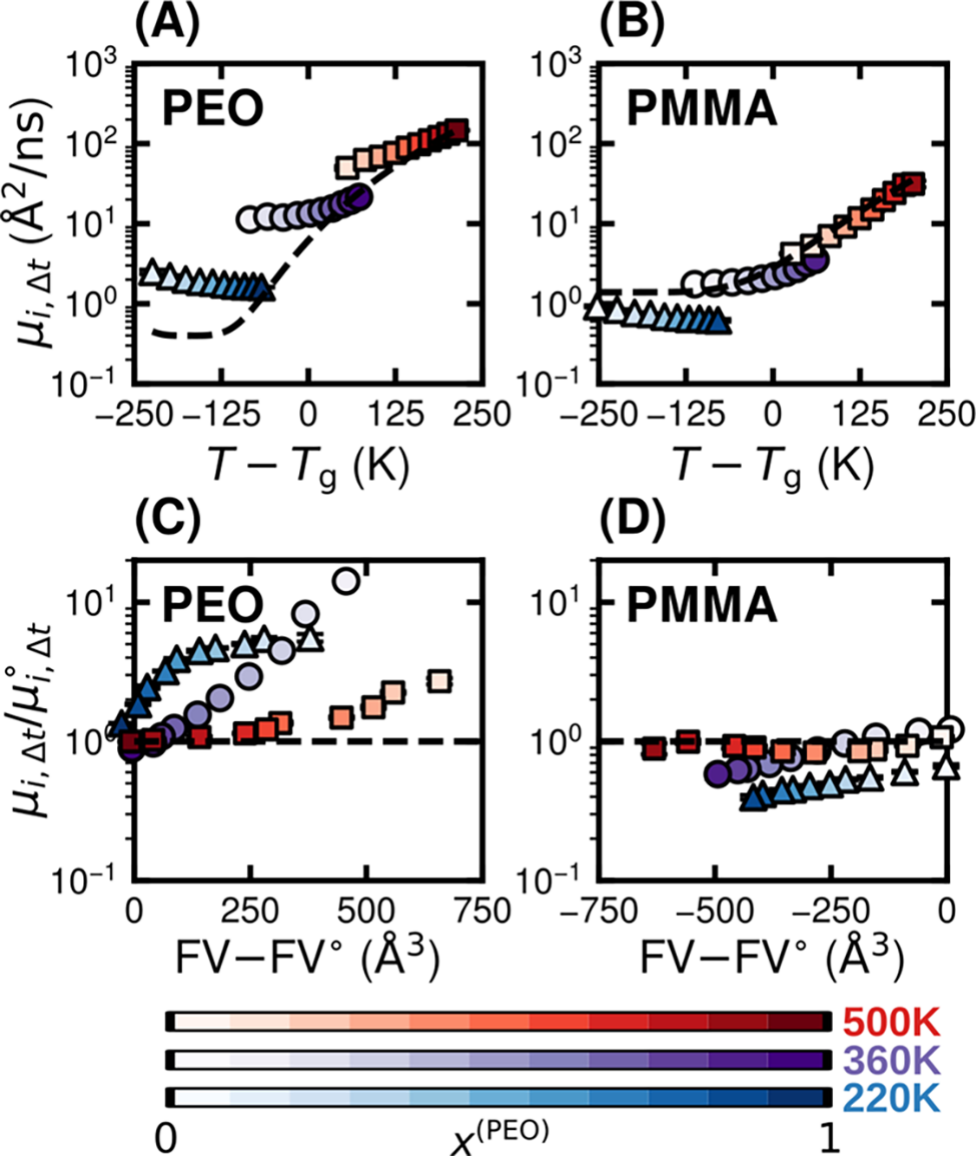
Analysis of local segmental mobilities across blend compositions and temperatures. The local segmental mobility, μ_*i*,Δ*t*_, as a function of temperature relative to the apparent *T*_g_ for (A) PEO and (B) PMMA. Data points at 220, 360, and 500 K are represented as blue triangles, purple circles, and red squares, respectively. The dashed black lines represent fits to the neat polymer reference, μ_*i*,Δ*t*_^°^. Segmental mobility as a function of free volume (FV) for (C) PEO and (D) PMMA. The FV° denotes reference to the FV of the neat polymer. Error bars reflecting standard errors from three independent systems are generally smaller than the symbol size.

To elucidate the prior results, we examine variations in species-dependent packing behavior, which is expected to manifest in different free volumes of the chains. Figure 2C,D shows the normalized segmental mobility as a function of a change in free volume (FV) from the neat system (denoted by ‘○’). The rationale for this comparison derives from considerations involving free-volume theory, which may suggest that log­[μ_*i*,Δ*t*_/μ_*i*,Δ*t*_^°^] ∝ (FV – FV°). The data indeed possess roughly linear behaviors in the limit of smaller perturbations in FV. The positive trends in Figure 2C,D across all temperatures indicate that positive deviations in segmental mobility from neat polymer behavior generally correlate with increases in local free volume upon blending and vice versa.

Increases in FV also explain the apparent enhancement in mobility of both PEO and PMMA at 220 K with increasing mole fraction of the lower mobility species. In particular, both species are effectively glassy at 220 K, with low mobilities of similar magnitude (μ_PEO,Δ*t*_^°^ = 0.16 Å/ns and μ_PMMA,Δ*t*_^°^ = 0.09 Å/ns), such that the relative mobility advantage of PEO is diminished (Figure S4). Consequently, in this regime, we suggest that the dynamics are mostly controlled by variations in free volume. Although PMMA is canonically slower, it is also bulkier, such that its addition leads to increased FV, and thus less effective packing. By contrast, PEO-rich blends exhibit lower mobilities due to more efficient packing of chains. The less effective packing of PMMA relative to PEO is supported by the observation that the free volume of neat PMMA surpasses that of neat PEO at the same *T* – *T*_g_ (Figure S3).

An exception to the observation that dynamics are controlled by free volume relates to PMMA at high temperatures. In this case, mobility in blends resembles that of a *T*_g_-equivalent neat polymer reference, despite variations in FV (Figure 2D). These trends support the notion that packing effects contribute to dynamic heterogeneity, but they again highlight an asymmetry in coupling. While increased free volume tends to correlate with enhanced mobility for PEO, a reduction in free volume does not universally imply suppressed segmental dynamics in PMMA by the same magnitude. This asymmetry reflects the influence of other factors beyond free volume in controlling relative enhancement/suppression of polymer dynamics in blends.

### 3.3 Influence of Local Environment on Segmental Mobility

#### 3.3.1 Analysis of Local Mobility-Composition Coupling

While previous results focused on local dynamics at the species level, we now explicitly consider variations due to the local environment of individual polymer segments. The central hypothesis is that a PEO segment surrounded entirely by other PEO segments should behave similarly to one in a pure PEO system, with minimal influence from PMMA, and vice versa. However, as the local environment becomes enriched in the opposite species, there will be interaction-based coupling that will lead to deviations. To test this, we analyze segmental mobilities as a function of a normalized self-density parameter ϕ̃_*i*_^(*A*)^, which is approximately unity when the environment is similar to that of the neat system and approaches zero when surrounded completely by the other species.

Figure 3 shows that the influence of local environment is asymmetric between species and strongly temperature-dependent. The temperature-dependence is first apparent by comparing the results for PEO where μ_*i*,Δ*t*_ is enhanced by blending at a temperature below both component *T*_g_ (Figure 3A) whereas it is suppressed upon blending at higher temperatures (Figure 3B,C). Meanwhile, that the influence is asymmetric between species is evident by contrast with the PMMA results (Figure 3D–F), which display nominally the opposite behavior of enhancement upon blending at high temperatures and minor suppression upon blending at low temperatures. The observations at 360 and 500 K are physically intuitive, as PEO-rich environments tend to exhibit larger mobilities, while PMMA-rich environments are slower. The observations at 220 K where mobilities appear enhanced in PMMA-rich environments are likely due to the free-volume effects described in Section 3.2.

**Figure 3:**
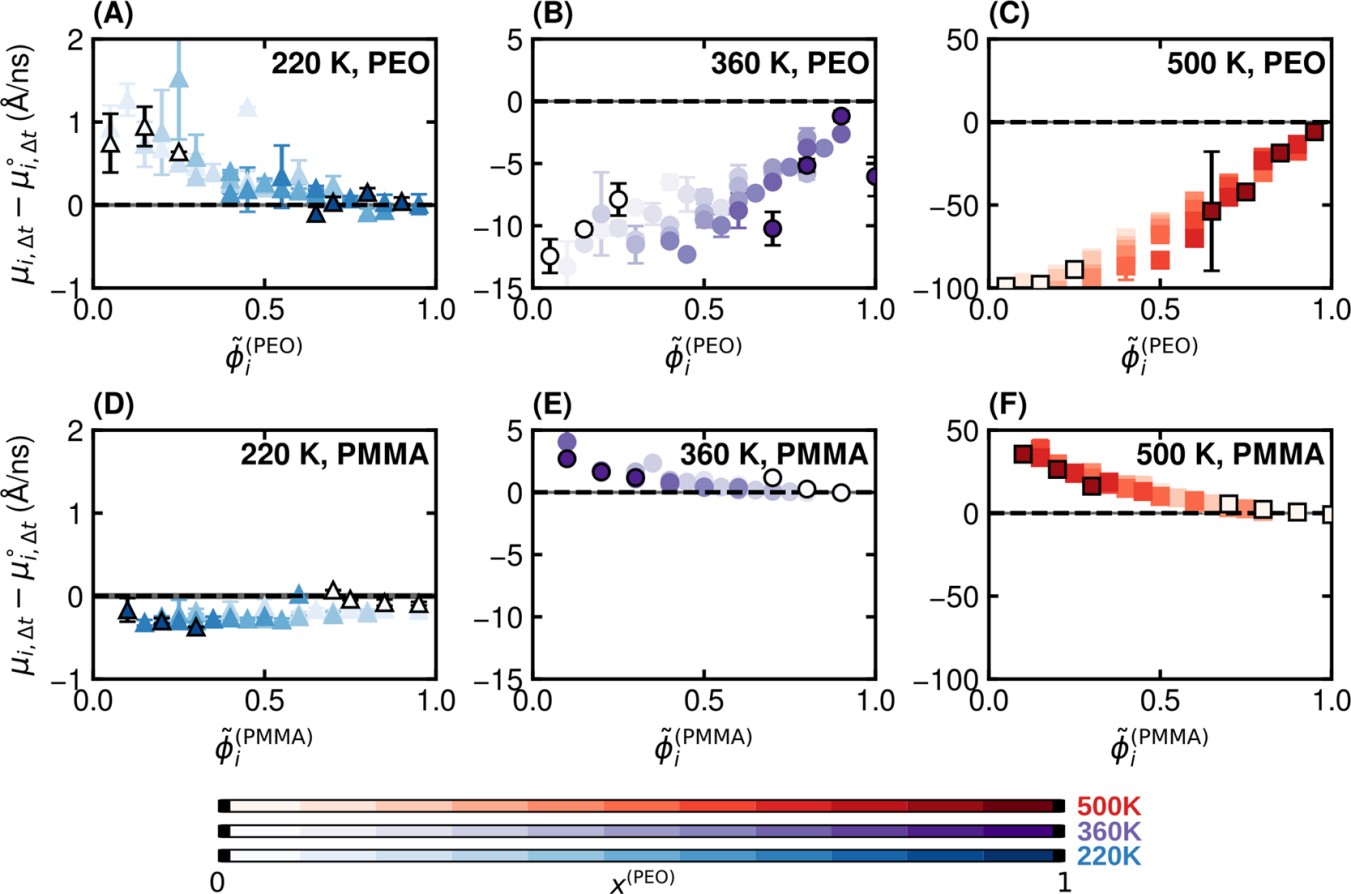
Variation in relative segmental mobility based on local environment. Deviations for neat-polymer mobility for PEO at (A) 220 K, (B) 360 K, and (C) 500 K and for PMMA at (D) 220 K, (E) 360 K, and (F) 500 K. Data is shown for all blend compositions, with gradation from light (PMMA-rich) to dark (PEO-rich), as indicated by the color bars. Results for chains in blends with the most extreme compositions (*x* ^(PEO)^ = 0.1 and 0.9) are outlined in black for visual clarity. Error bars reflect standard errors from three independent systems. Horizontal dashed lines provide a guide to the eye for the neat-polymer mobility. The gray shaded area around the dashed lines reflect standard deviations calculated from three independent neat systems.

The dependence of segmental mobility on local composition also displays intriguing differences between suppression and enhancement effects in blends. In cases where μ_*i*,Δ*t*_ is enhanced upon blending (above the dashed line), μ_*i*,Δ*t*_ gradually approaches the neat reference, μ_*i*,Δ*t*_^°^, as the local environment becomes enriched in the same species; the notion of gradual in this context reflects a vanishing of the first derivative, $\frac{\partial \mu_{i,\Delta t}}{\partial {\tilde{\phi }}_{i}}$ . Where μ_*i*,Δ*t*_ is suppressed (below the dashed line), μ_*i*,Δ*t*_ approaches μ_*i*,Δ*t*_^°^ more abruptly. While all dynamics tend to the neat reference in the limit that the local environment is enriched in that species, these behaviors, which are species-agnostic, reveal that suppression effects are more readily evident than enhancements. In other words, the local coupling of polymers with the opposing species has a much stronger magnitude of effect whereby the mobility of faster-moving chains is reduced more than that of slower-moving chains is enhanced in a blend. Upon investigation of the behavior of μ_*i*,Δ*t*_ – μ_*i*,Δ*t*_^°^ normalized by μ_*i*,Δ*t*_^°^ shown in Figure S5, it is evident, however, that the relative change in μ_*i*,Δ*t*_ is actually much larger for species with enhanced mobility than suppressed mobility.

There is also disparity in the composition-dependence of these observations between the two polymer species. In PEO, Figure 3B,C shows that trends in segmental mobility with respect to ϕ̃_*i*_^(PEO)^ differ based on *x* ^(PEO)^. We observe that the extent of dynamical coupling is weaker in blends with more PMMA (i.e., for a given ϕ̃_*i*_^(PEO)^, μ_*i*,Δ*t*_ – μ_*i*,Δ*t*_^°^ is smaller in systems with lower *x* ^(PEO)^). By contrast, in PMMA, Figure 3E,F shows how μ_*i*,Δ*t*_ – μ_*i*,Δ*t*_^°^ collapses onto a single curve for all compositions. This can be accounted for by the composition-dependent packing behavior. Namely, the suppression of PEO mobility due to the presence of more PMMA in the local environment is negated in part by the larger free volume, which would tend to enhance mobility of chains in PMMA-rich blends (Figure 2). This effect is largely absent for PMMA at all temperatures, once again reflecting the asymmetrical nature of dynamic heterogeneity in these blends.

#### 3.3.2 Analysis of Local Composition Distributions

While Figure 3 demonstrates how local composition leads to deviations in segmental dynamics from neat-polymer behavior, further understanding blend properties requires knowledge of how frequently such environments occur and how they are distributed. To this end, we characterize the local environments sampled by PEO and PMMA with the objective to relate these to deviations from *T*_g_-equivalent neat dynamics. We compute probability distributions of local intermolecular PMMA volume fractions surrounding particles from PEO, ϕ_inter,PMMA_^(PEO)^, and PMMA, ϕ_inter,PMMA_^(PMMA)^, and characterize their breadth using a normalized Shannon entropy, *H̃*, where lower *H̃* indicates more homogeneous environments and higher values indicates environments are more heterogeneously distributed.

Figure 4A shows how the distributions of intermolecular PMMA volume fractions around PEO units vary with blend composition at 500 K; analogous results at 220 and 360 K are provided in the Supporting Information (Figure F8). For PEO-rich blends, the distributions peak near ϕ_inter,PMMA_^(PEO)^ = 0, indicating that most PEO atoms experience negligible local PMMA content, consistent with the overall scarcity of PMMA. As *x* ^(PEO)^ decreases and there is more PMMA present in the blend, the distributions shift toward higher ϕ_inter,PMMA_^(PEO)^; the average intermolecular PMMA volume fraction tracks neatly with overall blend composition (see progression of stars in Figure 4A and Figure S11A). However, the distributions also broaden, revealing that local environments become increasingly heterogeneous as the blend composition becomes more balanced. This trend is reflected quantitatively in the normalized Shannon entropy, *H̃* (Figure 4B), which rises with decreasing *x* ^(PEO)^, signifying a wider range of local PMMA fractions sampled by PEO atoms. The entropy plateaus for *x* ^(PEO)^ ≲ 0.6, indicating that the extent of heterogeneity becomes composition-independent once sufficient PMMA is present in the blend.

**Figure 4:**
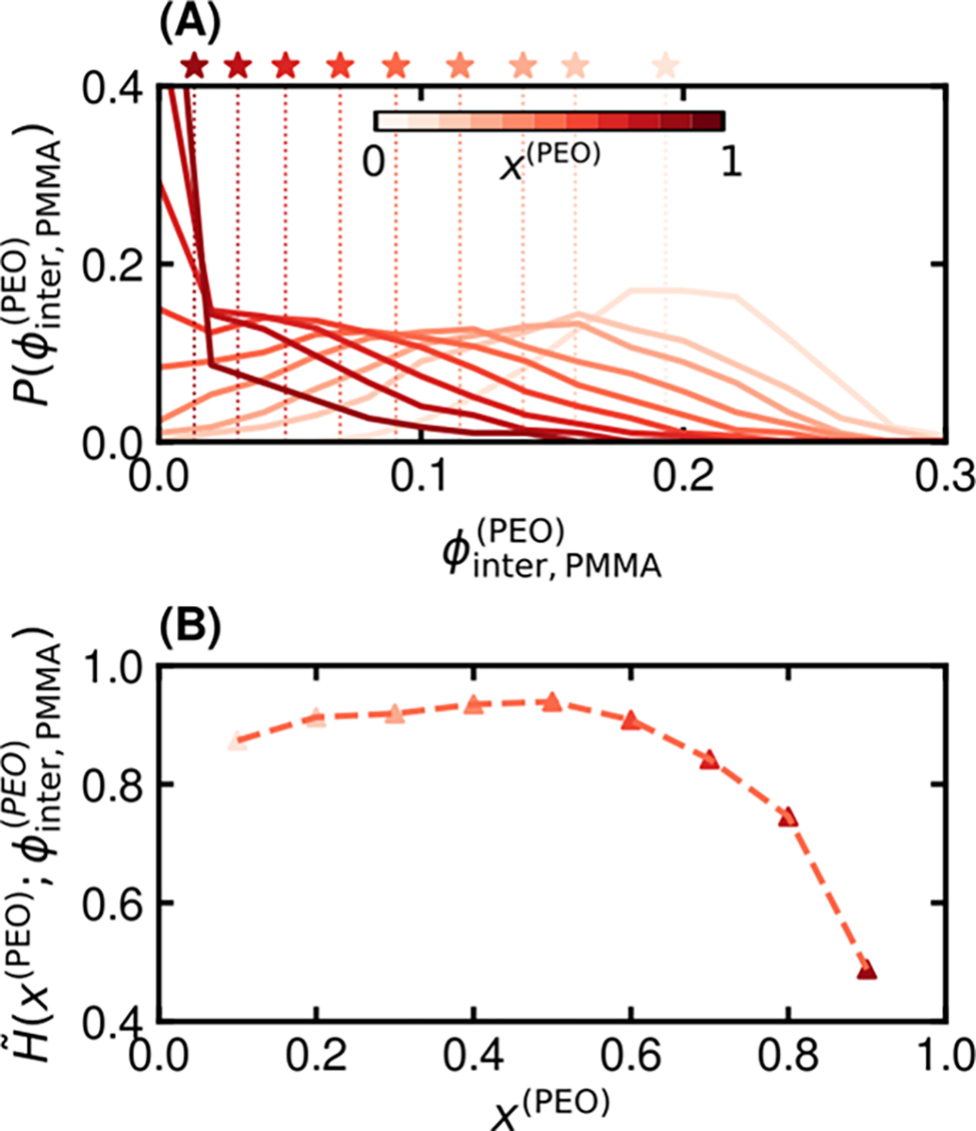
Characterization of local composition distributions around PEO. (A) Probability distributions of intermolecular PMMA volume fraction around PEO and (B) corresponding normalized Shannon entropies. Results are shown for systems at 500 K. The color gradient corresponds to a gradient in blend composition containing the most PEO (dark) to the least PEO (light). Star markers and corresponding vertical dotted lines in (A) indicate the average intermolecular PMMA volume fraction, ⟨ϕ_inter,PMMA_^(PEO)^⟩, with coloring to indicate the total blend composition. The dashed red line in (B) is provided as a guide to the eye.

Figure 5A shows how the distributions of intermolecular PMMA volume fractions around PMMA units vary with blend composition. As in Figure 4A, the distributions shift to higher ϕ_inter,PMMA_^(PMMA)^ with decreasing *x* ^(PEO)^ (progression of stars in Figure 4B and Figure S11B), reflecting the overall increase in PMMA content. However, unlike the distributions around PEO, their shapes remain comparatively narrow and composition-invariant, indicating that the local environments of PMMA change in a more uniform and predictable manner. This behavior is corroborated by the normalized Shannon entropy in Figure 5B, which remains nearly constant across compositions and is systematically lower than for PEO, signifying more homogeneous local surroundings. Together, these results highlight another asymmetry between PEO and PMMA upon blending. Namely, while the local environment of PMMA changes in a consistent way that can be tracked by the mean composition, as in the Lodge–McLeish framework,^48^ the local environment of PEO cannot because the nature of the distribution itself changes significantly with composition. In addition, PEO segments experience overall more local compositional heterogeneity.

**Figure 5:**
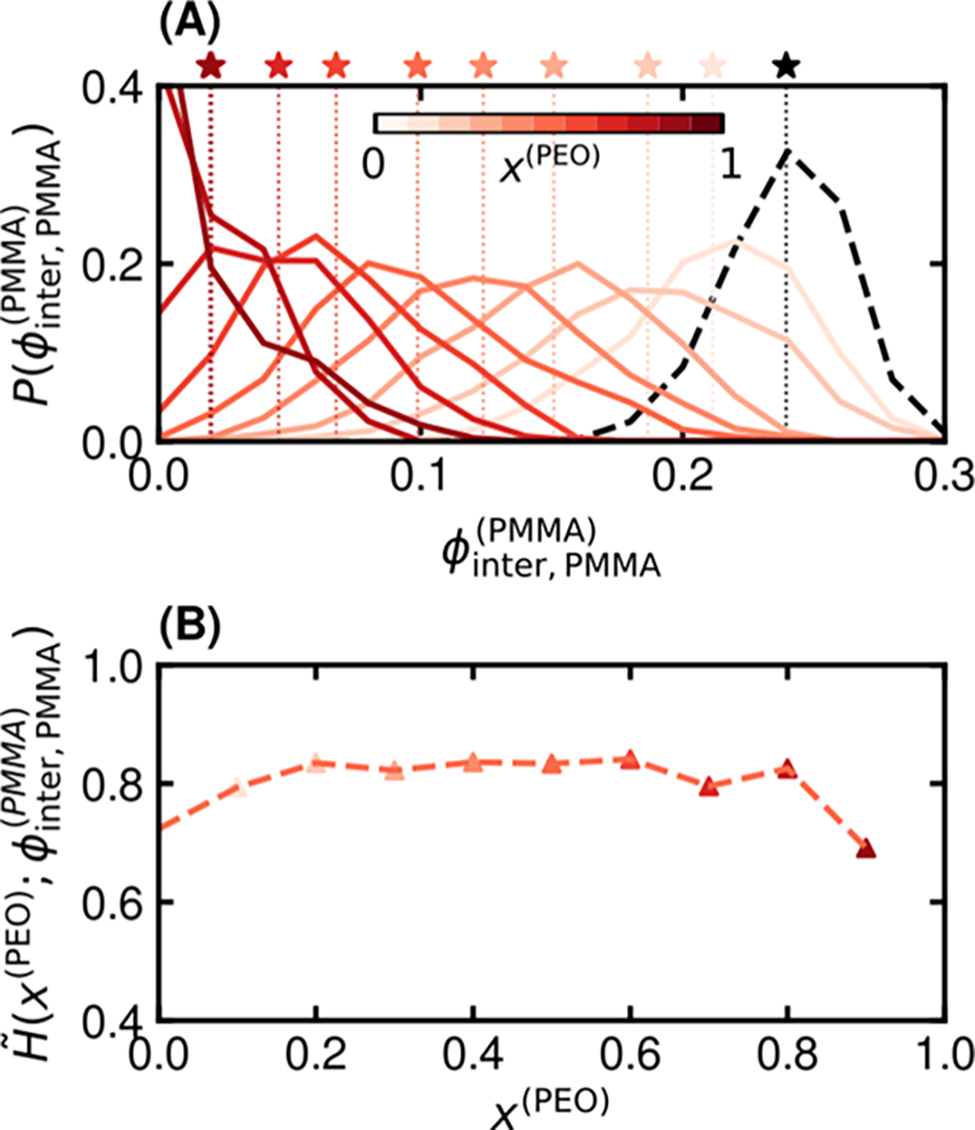
Characterization of local composition distributions for PMMA. (A) Probability distributions of intermolecular PMMA volume fraction around PMMA and (B) corresponding normalized Shannon entropies. Results are shown for systems at 500 K. The color gradient corresponds to a gradient in blend composition containing the most PEO (dark) to the least PEO (light). The black dashed line in (A) is the distribution of neat PMMA. Star markers and corresponding vertical dotted lines in (A) indicate the average intermolecular PMMA volume fraction, ⟨ϕ_inter,PMMA_^(PEO)^⟩, with coloring to indicate the total blend composition. The red dashed line in (B) is provided as a guide to the eye.

We attribute the dynamical response of PEO in the blends to the increased heterogeneity and composition dependence of its local environment with respect to surrounding PMMA. These features correlate strongly with the deviations of local segmental mobilities from those of *T*_g_-equivalent neat systems (Figure 2). For PEO, the growing diversity of local intermolecular PMMA environments with decreasing *x* ^(PEO)^ (Figure 4A) coincides with its marked departure from the neat reference behavior. In contrast, the local environments of PMMA evolve more uniformly with composition (Figure 5), resulting in dynamics that follow expectations based on the overall reduction in *T*_g_ with PEO incorporation. Distributions of local intramolecular self-contributions (Figure S6) are comparatively insensitive to composition, as expected. Meanwhile, the distributions of local intermolecular PEO contributions (Figure S7) again change simply in response to composition for PMMA, and for PEO, there is a modest increase in heterogeneity at low PMMA blending fractions, but this is less pronounced than the strong compositional diversity observed for surrounding PMMA (Figure S8). However, the distributions of local environments show minimal temperature dependence (Figures S6–S9), indicating that increased compositional heterogeneity alone does not account for the deviations observed well below *T*_g_ (220 K). Instead, at such temperatures, the relative dynamics are better explained by differences in free volume between the probed systems and their references (Figure 2C,D).

### 3.4 Analysis of Collective Segmental Relaxation in the Melt State

To gain insight into how blending affects collective segmental dynamics of PEO and PMMA, we compare the relaxation of Rouse mode coordinates computed using the positions of chemical monomers for chains of both species. In this context, the Rouse coordinates, computed by eq 6, provide a convenient collective variable to monitor how relaxation times vary with subchain length and composition. Thus, we aim to complement prior analysis on purely local segmental fluctuations with a description of subchain relaxations across longer times and length scales–not necessarily to test whether the dynamics are strictly Rouse-like. Given the computational challenges of equilibration and convergence as well as the dramatic increase in relaxation times expected at temperatures below *T*_g_, our analysis is restricted to systems at 500 K (Figure 6).

**Figure 6:**
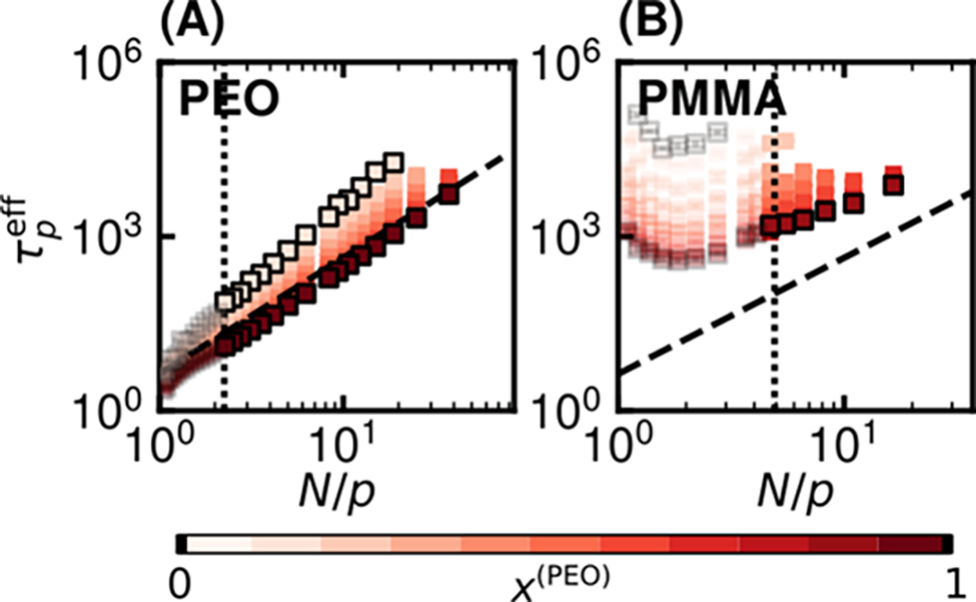
Rouse mode analysis at 500 K for chains in blends of varying composition. The effective Rouse relaxation time τ_*p*_^eff^ as a function of subchain length *N*/*p* for (A) PEO and (B) PMMA. For *p* > 8, symbols for data are only shown for every third value of *p* for visual clarity. Dashed black lines are a guide to the eye to indicate the expected ideal scaling of τ_*p*_ ∼ *p* ^–2^. The position of the line is the same across panels and is set to align with the behavior of neat PEO. Results for chains in blends with the most extreme compositions are outlined in black for visual clarity of trends. Error bars reflect standard errors from three independent systems and are generally smaller than the symbol size. Transparent markers are used for *N*/*p* corresponding to subchains equal to or less than an estimated Kuhn length. Dotted black vertical lines mark the *N*/*p* corresponding to a subchain of Kuhn length size for both PEO and PMMA, which is equal to the number of monomers per Kuhn step (see Table 1). Deviations from the ideal *p* ^–2^ scaling are expected for modes shorter than the Rouse bead size, which is generally larger than a Kuhn length.

Figure 6A shows that the characteristic relaxation times, τ_*p*_^eff^, of PEO in PEO/PMMA blends deviates from those of pure PEO, across all blend compositions and for all mode numbers. Pure PEO approximately follows the expected Rouse scaling (dashed black line), τ_*p*_ ∼ *p* ^–2^,^95^ albeit when using τ_*p*_^eff^. As the mode number increases (*p* → *N*), τ_*p*_^eff^ for all blends begins to converge; this implies that the relaxation behavior of the smallest subchains of PEO are similar, whether in blends or in neat melts.

It should be noted that the classic Rouse scaling of τ_*p*_ ∼ *p* ^–2^ is only expected down to the Rouse bead size, which is distinct from the Kuhn length and can span multiple chemical monomers.^96^ Here, the apparent convergence in relaxation times occurs for subchains likely smaller than a canonical Rouse bead. Meanwhile, deviations from pure PEO behavior become more pronounced at lower *p* as the blend becomes more PMMA-rich (decreasing *x* ^(PEO)^). Furthermore, since τ_*p*_^eff^ represents the mean relaxation time extracted from stretched exponential decays, apparent deviations from ideal scaling may reflect both physical departures^83–85^ from Rouse dynamics as well as nonexponential relaxation. All together, these results intuitively suggest that chain relaxation at shorter length scales is less affected by the surrounding melt environment than at longer length scales.

Figure 6B shows that variations in relaxation behavior of PMMA chain segments with *N*/*p* are functionally similar across blend compositions, resulting in systematic vertical offsets in relaxation times. This indicates that blending primarily shifts the overall time scale of PMMA dynamics while preserving their scaling, suggesting that the data might be collapsed by a composition-dependent rescaling factor, governed by the distance from *T*_g_ (see also Figure S21). Furthermore, the relaxation times for PMMA deviate from classical Rouse scaling across all mode numbers and compositions, exhibiting an upturn at short subchain lengths. This anomalous slowdown likely reflects that the relatively stiff PMMA chains contain only a few effective Rouse beads, such that the shortest modes are dominated by local constraints rather than entropic relaxation. This contrasts with Figure 6A, where PEO relaxation times scale differently with *N*/*p* as a function of blend composition yet converge for short subchains, indicating that PEO dynamics can recover neat-like behavior at sufficiently small length scales. In comparison, PMMA dynamics remain uniformly perturbed by PEO across all scales.

### 3.5 Interplay between Local Mobility and Relaxation Time Scales

At this stage, we remark on an apparent contradiction between the results shown in Figure 6A,B and those in Figure 3C,F. Figure 3 indicates that PEO segmental mobility is suppressed even in the presence of a small number of nearby PMMA segments, whereas PMMA mobility is only notably affected when the local environment becomes significantly enriched in PEO (ϕ̃_*i*_^(PMMA)^ < 0.5). In contrast, the Rouse mode analysis suggests a strong overall influence of PEO on PMMA because relaxation times for PMMA do not converge to the neat reference, even for short subchains, whereas those for PEO do.

Two factors help reconcile this discrepancy. First, although PEO appears more affected in absolute terms in Figure 3, normalizing by the neat-polymer reference reveals that PMMA experiences a much larger relative enhancement in dynamics in PEO-rich environments (Figure S5). Second, there is also a fundamental distinction between the two measured quantities. Figure 3 relates to a segmental mobility, based on short-time fluctuations over a 100 ps interval, and Figure 6 relates to segmental relaxation, which reflects structural decorrelation and occurs at much longer time scales. Thus, while local dynamics may appear similar over short times, this does not necessarily imply similar behavior in longer-time relaxation. We suspect that results between these analyses would begin to align if the time interval for assessing segmental mobility was substantially increased. This underscores a key nuance of dynamic heterogeneity, in that its effects depend on the time scale of the process being observed.

## 4 Conclusions

The seemingly straightforward dependence of PMMA dynamics on a shifted *T*_g_ in contrast to the composition-dependent dynamics of PEO in PEO/PMMA blends have been the subject of several prior works.^40–43^ Here, using atomistic molecular dynamics simulations, we extend these observations by systematically examining how overall and local composition influence segmental mobility and collective relaxation, across blend compositions, multiple length scales, and thermal regimes. Analyses of both local fluctuations and subchain relaxation dynamics reveal a coherent physical picture linking free-volume effects and local compositional heterogeneity to deviations from neat-polymer behavior.

Local environments were found to asymmetrically affect segmental mobilities. At short times, suppression by “slow” environments was stronger than enhancement by “fast” ones; nonetheless, PEO accelerated PMMA dynamics far more than PMMA suppressed PEO. Accounting for *T*_g_ differences, PEO segments in blends exhibited enhanced dynamics relative to neat PEO, whereas PMMA segments in blends remained comparable to *T*_g_-equivalent neat PMMA systems. These effects were directly connected to variations in local composition. PEO dynamics increased with local free volume, which is heterogeneously distributed when blending with PMMA, yielding a response that differs from mean-field expectations. Meanwhile, PMMA dynamics varied more uniformly with blend composition, as does the local compositional variations of PMMA segments.

Rouse-mode analysis at 500 K revealed complementary signatures at collective length scales. PEO relaxation approached neat-like behavior at short subchain lengths or in PEO-rich blends, whereas PMMA relaxation was uniformly accelerated across all modes with increasing PEO content. This behavior suggests a more idealized, composition-dependent response for PMMA compared to the strongly heterogeneous PEO dynamics. In tandem with our observations on the local enhancement of PMMA dynamics in the vicinity of PEO, we suggest this arises from a nanoscale facilitation mechanism whereby locally softened PMMA near PEO propagates enhanced mobility to neighboring regions. This interpretation resonates with the dynamic coupling framework proposed by Ngai and Roland,^97^ in which mobility in the faster component propagates through intermolecular interactions to accelerate relaxation of the slower matrix.

Overall, these findings provide a molecular-level framework for understanding dynamic asymmetry in polymer blends. We note that our results pertain to amorphous systems of syndiotactic PMMA. Given the similar *T*_g_ and segmental dynamics of atactic PMMA, we expect our findings to be broadly transferable to amorphous blends containing atactic PMMA as well. The same analytical approaches could be applied to systems with crystallinity or other tacticities to probe how packing and morphology shape asymmetric dynamics. Similar mechanisms are anticipated in other flexible–rigid polymer pairs such as PDMS/PMMA or PDMS/PS, offering a basis to test the generality of this physical picture. More broadly, the results suggest that the influence of one species emerges only at certain local compositions and evolves nonlinearly with environment. This insight may guide strategies for tuning viscoelasticity or transport by adjusting the composition and morphology of dynamically distinct phases.

## Supplementary Material



## Data Availability

The data underlying this study are available in the published article and its Supporting Information. Additional files facilitating reproduction by performing simulations are available at https://github.com/webbtheosim/md-simulation-files/tree/main/2025-peo-pmma-blends.

## Notes

Calculation of apparent *T*_g_; calculation of free volume; comparison of neat PEO and neat PMMA free volume; relative segmental mobility enhancement as a function of temperature; influence of local environment on normalized segmental mobility; influence of the distribution of local environment on segmental mobility; and Rouse mode analysis (PDF)
