# Supplementary material for: Asymmetric Effects Underlying Dynamic Heterogeneity in Miscible Blends of Poly(methyl methacrylate) with Poly(ethylene oxide)
Source: Macromolecules. 2026 Feb 2;59(4):2302–14. doi: 10.1021/acs.macromol.5c01587 (PMC12947691; doi:10.1021/acs.macromol.5c01587)
Supplement: Supplementary file 1 [file ma5c01587_si_001.pdf]

Supporting Information

Asymmetric Effects Underlying Dynamic Heterogeneity in Miscible Blends of Poly(methyl methacrylate) with Poly(ethylene oxide)

Shannon Zhang and Michael A. Webb*

Department of Chemical and Biological Engineering, Princeton University, Princeton, NJ 08544, USA

E-mail: mawebb@princeton.edu

Contents

S1 Calculation of apparent T_g **SI-2**

S2 Calculation of free volume **SI-4**

S3 Comparison of neat PEO and neat PMMA free volume **SI-5**

S4 Relative segmental mobility enhancement as a function of temperature **SI-6**

S5 Influence of local environment on normalized segmental mobility **SI-7**

S6 Influence of the distribution of local environment on segmental mobility **SI-8**

S7 Rouse mode analysis **SI-15**

S1 Calculation of apparent T_g

The simulated T_g is calculated using a bootstrapping process that is repeated 10,000 times. The start and end points of the high and low temperature regions selected for each bootstrapping process are given in Table S1. This procedure will only yield a single apparent T_g representative of the system; different fitting procedures would be required in an attempt to identify multiple T_g signatures. The simulated specific volume versus temperature data used in this calculation, final T_g values, and example linear regressions in the high and low temperature regions for all blends studied are given in Figure S1.

Table S1: Start and end points and ranges of temperature used to calculate linear regressions in the high and low temperature regions of specific volume versus temperature data for T_g calculations of PEO/PMMA blends.

Composition	Glassy region fit	Liquid region fit
100 PEO%	100-[150,250]	[300,400]-450
90 PEO%	100-[150,250]	[350,450]-500
80 PEO%	100-[150,250]	[350,450]-500
70 PEO%	100-[150,250]	[400,500]-550
60 PEO%	100-[150,250]	[400,500]-550
50 PEO%	150-[200,300]	[400,500]-550
40 PEO%	150-[200,300]	[400,500]-550
30 PEO%	150-[200,300]	[440-540]-590
20 PEO%	200-[250,350]	[440-540]-590
10 PEO%	200-[250,350]	[500,600]-650
0 PEO%	200-[250,350]	[500,600]-650

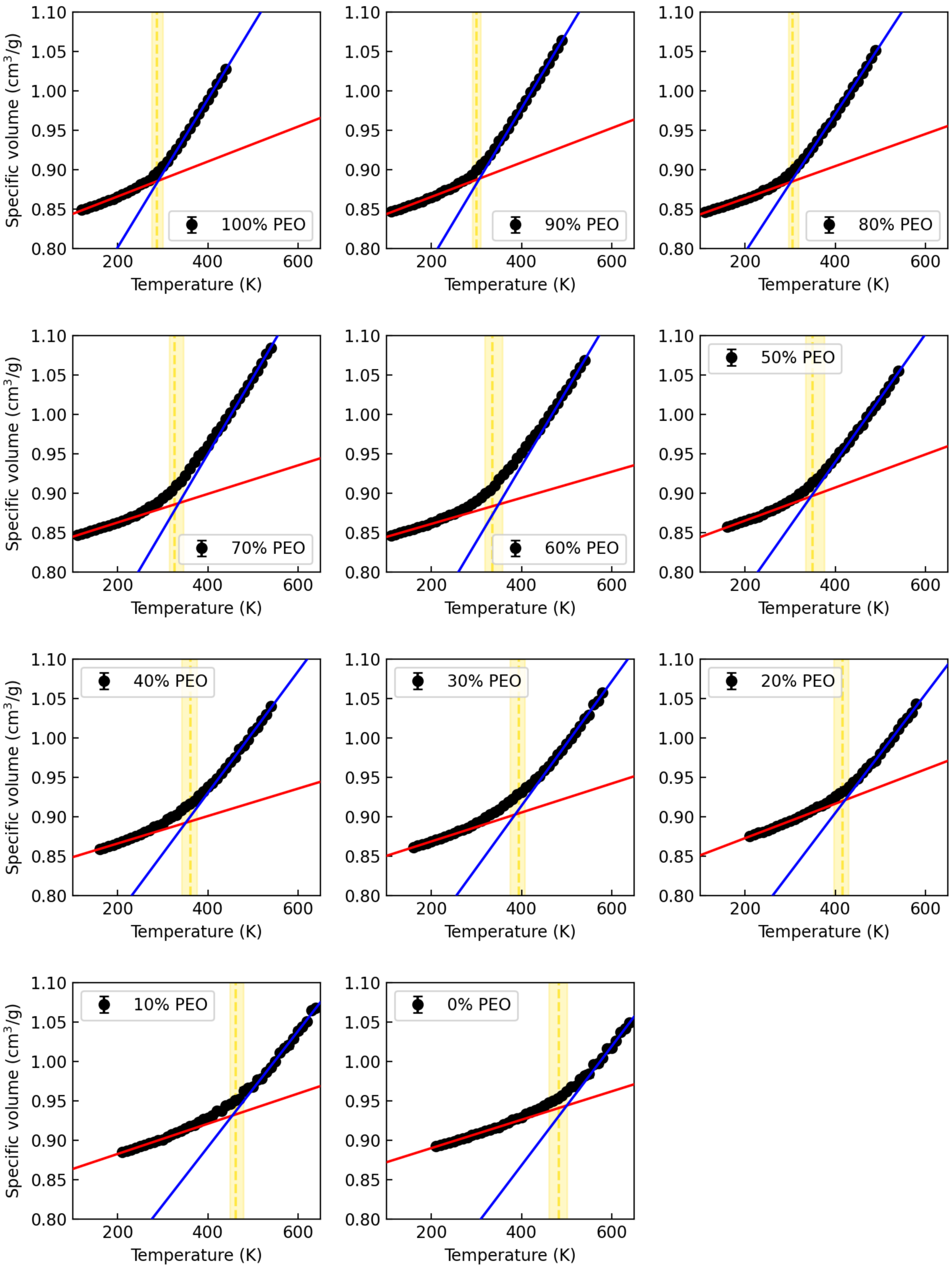

Figure S1: Change in specific volume with temperature used to calculate T_g for PEO/PMMA blends over blend composition. Each marker and error bar is an average and standard error, respectively, of the specific volume of three independent system configurations at a given temperature. The vertical dashed yellow line is the final calculated T_g. The shaded yellow region is the range of all T_g values calculated in the bootstrapping process. The red and blue lines are example linear regressions of one instance of the bootstrapping process for the high and low temperature regions, respectively.

S2 Calculation of free volume

The size of the probe used in the free volume calculation dictates the magnitude of free volume calculated for a polymer chain. Probes that are too large will underestimate free volume while probes that are too small will overestimate. However, we are interested in the qualitative change in free volume with x_{PEO}. To ensure that probe size does not affect our analysis, we calculated free volume using probes of radius 0.25 Å and 0.5 Å. These values are smaller than the size of the smallest atom in the polymer, hydrogen, which has a $\sigma = 1.008$ Å. As shown in Figure S2, we observe that adjusting the probe size affects the magnitude of free volume surrounding the chain, as expected, but does not change the qualitative trends in free volume versus x_{PEO}. Thus, using any of these probe sizes would be adequate for the analysis presented in the main text. We proceed using probes of radius 0.5 Å.

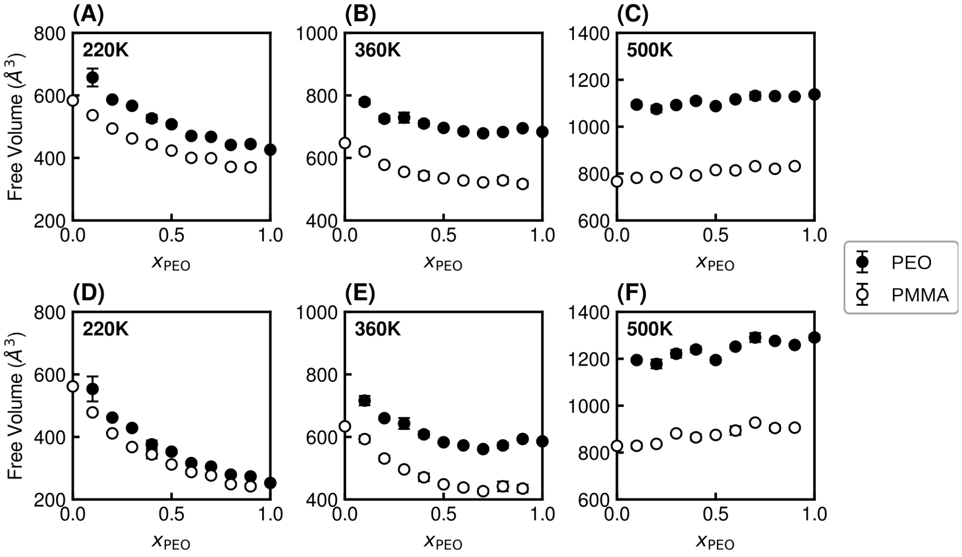

Figure S2: Local free volume of PEO and PMMA chains versus blend composition calculated using different probe radii. Free volume is calculated using probes of radius (A-C) 0.25 Å and (D-F) 0.5 Å. Error bars are standard errors calculated for three independent system configurations.

S3 Comparison of neat PEO and neat PMMA free volume

Changes in segmental mobilities of PEO and PMMA upon blending directly correlate with changes in free volume. We suggest that the unexpected behavior of segmental mobility at 220 K, where mobility for both PEO and PMMA decrease in blends with more PEO despite PEO being the more mobile polymer, is caused by the mobilities of both species being comparably low at 220 K and the less efficient packing of PMMA compared to PEO. We show that PMMA packs less efficiently than PEO in Figure S3, where the free volume per chain of neat PMMA is confirmed to be greater than the free volume per chain of neat PEO at all values of $T - T$g.

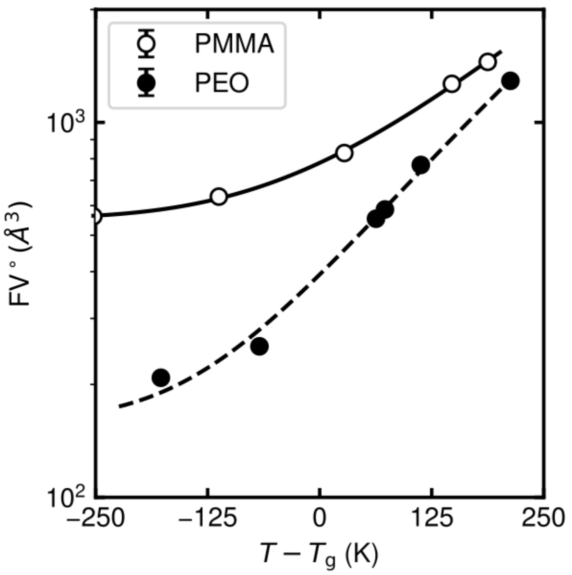

Figure S3: Local free volume of neat PEO and neat PMMA chains versus $T - T_g$. Free volume is calculated using probes of radius 0.5 Å. The solid black line represents a fit to the neat PMMA free volume and the dashed line represents a fit to the neat PEO free volume. Error bars are included as standard errors calculated for three independent system configurations but are generally smaller than the symbol size.

S4 Relative segmental mobility enhancement as a function of temperature

Local segmental mobility as a function of temperature in Section 3.2 reveals unexpected behaviors at 220 K which oppose the behaviors at 360 K and 500 K. This is due to the low relative mobility of PEO over PMMA at 220 K (below the blend T_g). As such, the dominant factor controlling dynamics is free volume. At 360 K and 500 K, the relative mobility of PEO over PMMA is much higher, as shown in Figure S4.

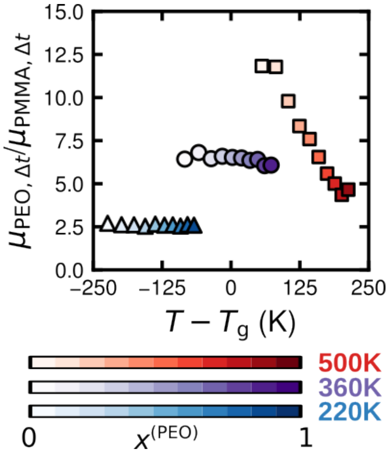

Figure S4: Relative mobility enhancement of PEO over PMMA as a function of temperature. The color gradient corresponds to a gradient in blend composition containing the most PEO (dark) to the least PEO (light). Error bars are standard errors for three independent system configurations at a given composition and temperature and are generally smaller than the symbol size.

S5 Influence of local environment on normalized segmental mobility

Analysis of the segmental mobility as a function of local mobility in Section 3.3.1 reveals that changes to the local environment lead to larger magnitudes of $\mu_{i,\Delta t}$ suppression than $\mu_{i,\Delta t}$ enhancement. However, $\mu_{i,\Delta t}$ normalized by the local mobility of each neat species ($\mu^{\circ}_{i,\Delta t}$), shown in Figure S5, reveals that the local environment actually has a much larger relative effect on the enhancement of normalized $\mu_{i,\Delta t}$ than the suppression of normalized $\mu_{i,\Delta t}$.

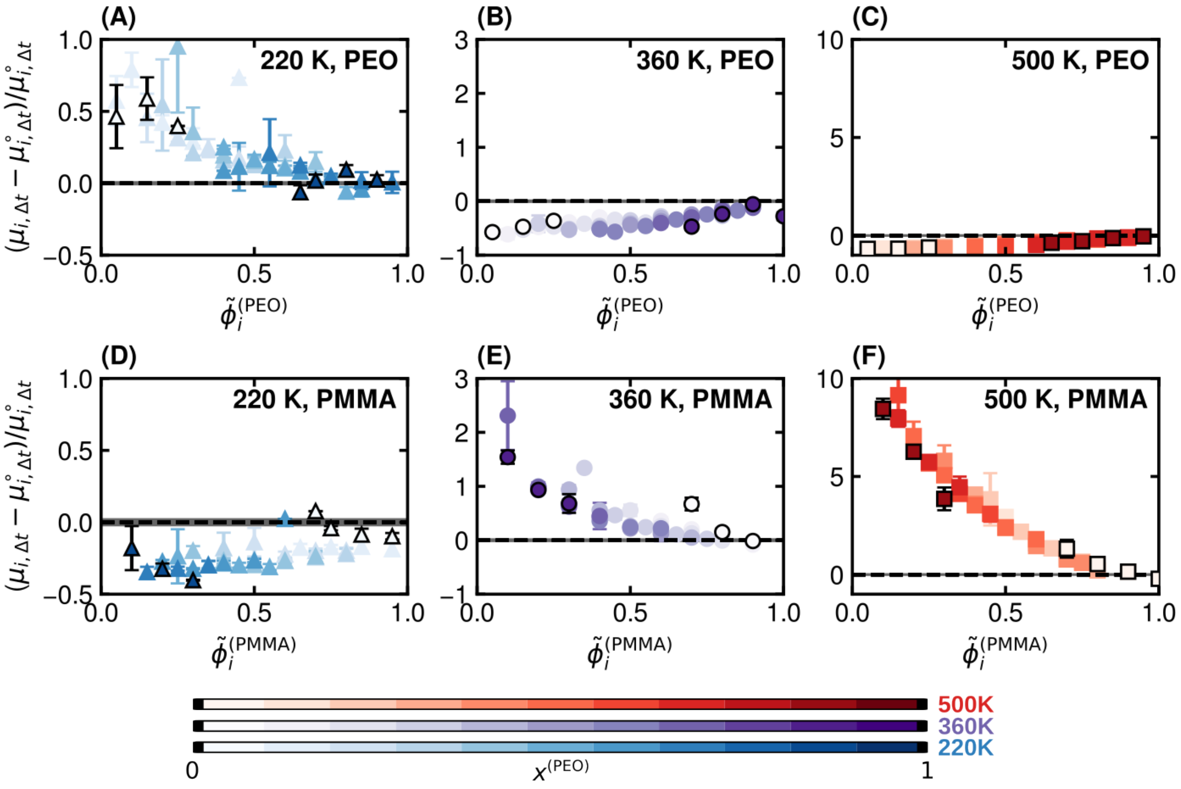

Figure S5: Variation in normalized relative segmental mobility over local environment. Results are shown for PEO at (A) 220 K, (B) 360 K, and (C) 500 K and PMMA at (D) 220 K, (E) 360 K, and (F) 500 K. Segmental mobility is calculated relative to and normalized by $\mu_{i,\Delta t}$ of the neat polymers, denoted as $\mu^{\circ}_{i,\Delta t}$. The color gradient corresponds to a gradient in blend composition containing the most PEO (dark) to the least PEO (light). Results for chains in blends with the most extreme compositions ($x^{(\mathrm{PEO})} = 0.1$ and 0.9) are outlined in black as a visual aid. Error bars are standard errors for three independent system configurations at a given composition and temperature. Horizontal dashed lines correspond to the relative segmental mobility of (A-C) pure PEO and (D-F) pure PMMA. The gray area around the dashed lines are standard deviations calculated from three independent configurations of the neat systems.

S6 Influence of the distribution of local environment on segmental mobility

The distribution of local environment is found to correlate with the change in $\mu_{i,\Delta t} - \mu^{\circ}_{i,\Delta t}$. This is concluded by analyzing the distribution of intermolecular PMMA around PEO and PMMA and its normalized Shannon entropy ($\tilde{H}$) at 500 K in Section 3.3.2. We have additionally calculated the distributions of intramolecular units and intermolecular PEO around PEO and PMMA at 220 K, 360 K, and 500 K, along with the distribution of intermolecular PMMA at 220 K and 360 K, and their normalized Shannon entropies.

Distributions of intramolecular PEO and PMMA are shown in Figure S6 and their $\tilde{H}$ in Figure S9A-B. All intramolecular distributions are found to be temperature and blend composition invariant. This is expected due to the small size of the cooperative sphere used in these calculations (the radii of the spheres are Kuhn lengths). Distributions of intermolecular PEO are shown in Figure S7 and their $\tilde{H}$ in Figure S9C-D. Although these distributions exhibit similar behaviors to the distributions of intermolecular PMMA that are discussed in Section 3.3.2, the diversity in local PEO around PEO is less than the diversity in local PMMA around PEO, particularly at low $x^{(\mathrm{PEO})}$. We conclude this because the distributions in S7A-C are more narrow than the distributions in S8A-C. This is also evident from the low values of $\tilde{H}$ for low $x^{(\mathrm{PEO})}$ observed in Figure S7. We attribute the reduction in distribution diversity to the asymmetric influence of local environment that are echoed throughout the text, wherein proximity to PMMA induces stronger dynamical changes than proximity to PEO. Distributions of intermolecular PMMA are shown in Figure S8 and their $\tilde{H}$ are shown in Figure S9E-F. Results are shown to be independent of temperature, as stated in Section 3.3.2.

Distributions are calculated for all figures in the text and SI for backbone carbons on both PEO and PMMA. Characterization of the composition around other atoms or collections of atoms on each polymer may result in different results. For PEO, we do not expect remarkable

differences between the composition parameters computed using carbon versus oxygen, since they are locally correlated within their bond distance. For PMMA, while we still expect the two backbone carbons to yield similar results, again due to local correlation, the atoms on the side chain could be different. Using different reference sites on PMMA, we show in Figure S10 that the composition around the side chain carbon is identical to that around the backbone carbons. Thus, the trends analyzed in Section 3.3.2 are robust.

Finally, we observe in Figures 4 and 5 that distributions shift toward higher intermolecular PMMA volume fractions with decreasing $x^{(\mathrm{PEO})}$. To highlight this trend, we have directly plotted the average intermolecular volume fractions against the blend composition in Figure S11.

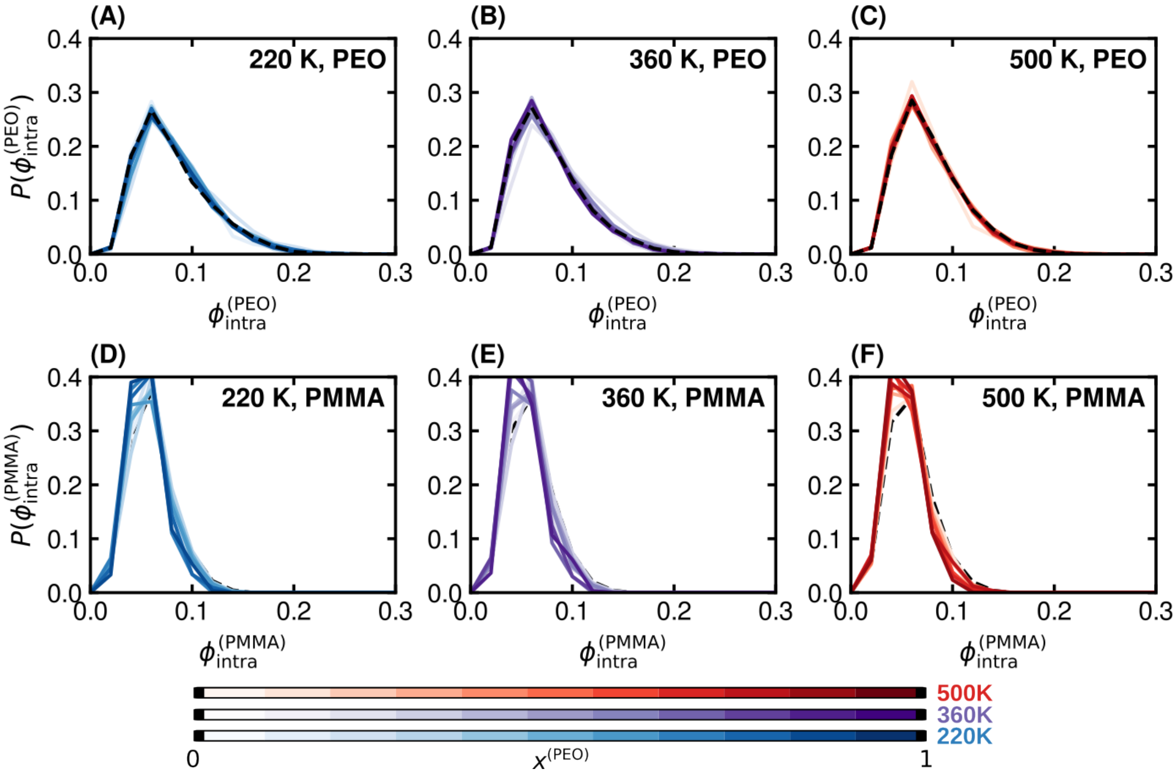

Figure S6: Probability distributions of the volume fraction of intramolecular (A,B,C) PEO and (D,E,F) PMMA atoms within a Kuhn length of each backbone carbon. Results are shown for PEO at (A) 220 K, (B) 360 K, and (C) 500 K and PMMA at (D) 220 K, (E) 360 K, and (F) 500 K. The color gradient corresponds to a gradient in blend composition containing the most PEO (dark) to the least PEO (light). Results for pure systems are shown as black dashed lines.

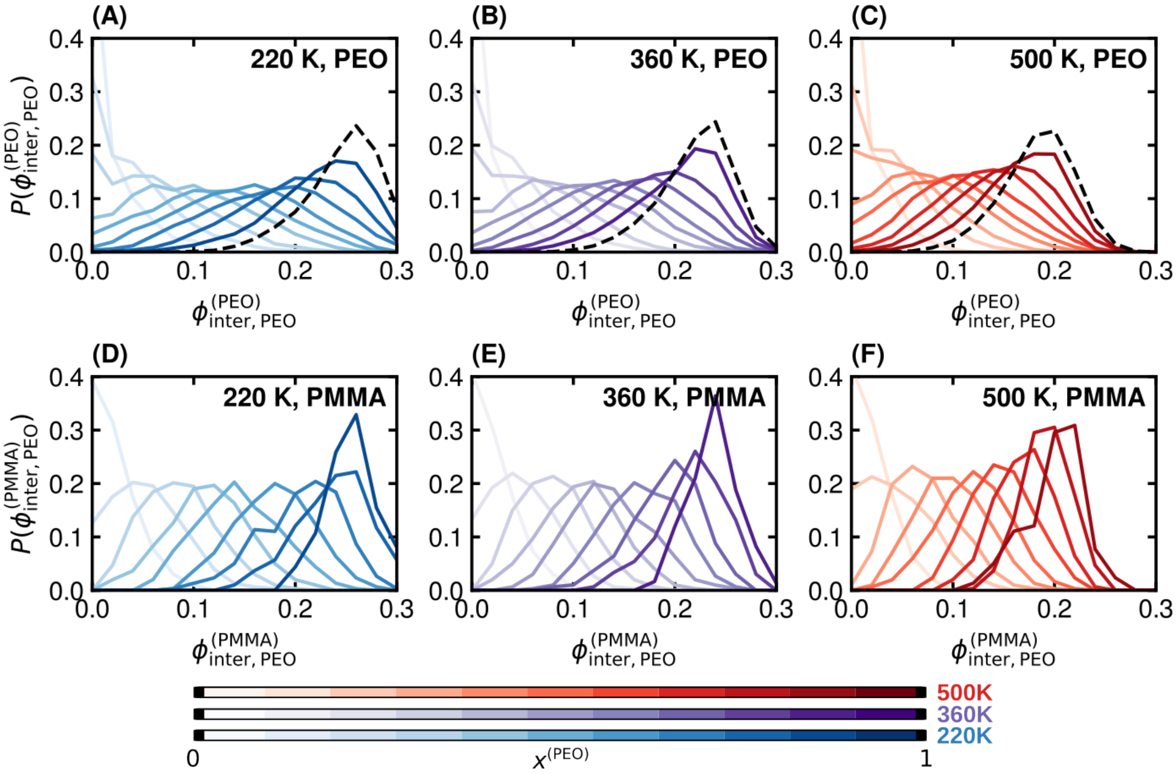

Figure S7: Probability distributions of the volume fraction of intermolecular PEO within a Kuhn length of backbone carbons on (A,B,C) PEO chains and (D,E,F) PMMA chains. Results are shown for PEO at (A) 220 K, (B) 360 K, and (C) 500 K and PMMA at (D) 220 K, (E) 360 K, and (F) 500 K. The color gradient corresponds to a gradient in blend composition containing the most PEO (dark) to the least PEO (light). Results for pure systems are shown as black dashed lines.

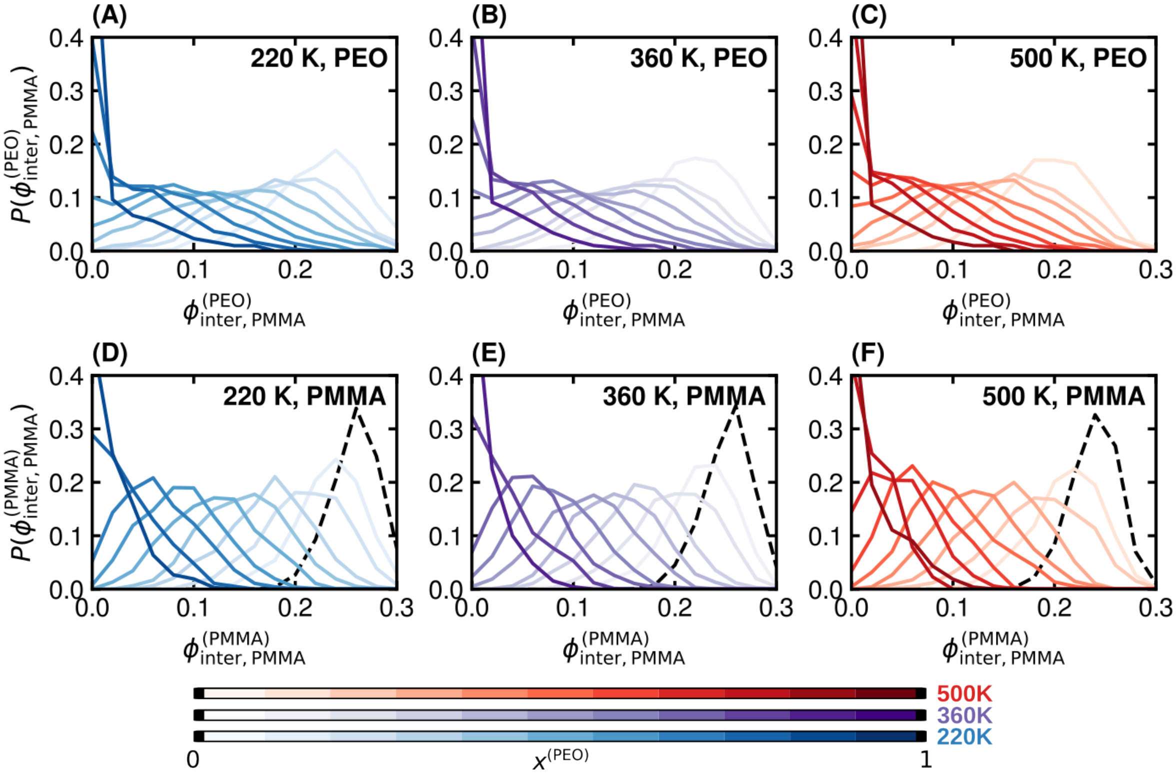

Figure S8: Probability distributions of the volume fraction of intermolecular PMMA within a Kuhn length of backbone carbons on (A,B,C) PEO chains and (D,E,F) PMMA chains. Results are shown for PEO at (A) 220 K, (B) 360 K, and (C) 500 K and PMMA at (D) 220 K, (E) 360 K, and (F) 500 K. The color gradient corresponds to a gradient in blend composition containing the most PEO (dark) to the least PEO (light). Results for pure systems are shown as black dashed lines.

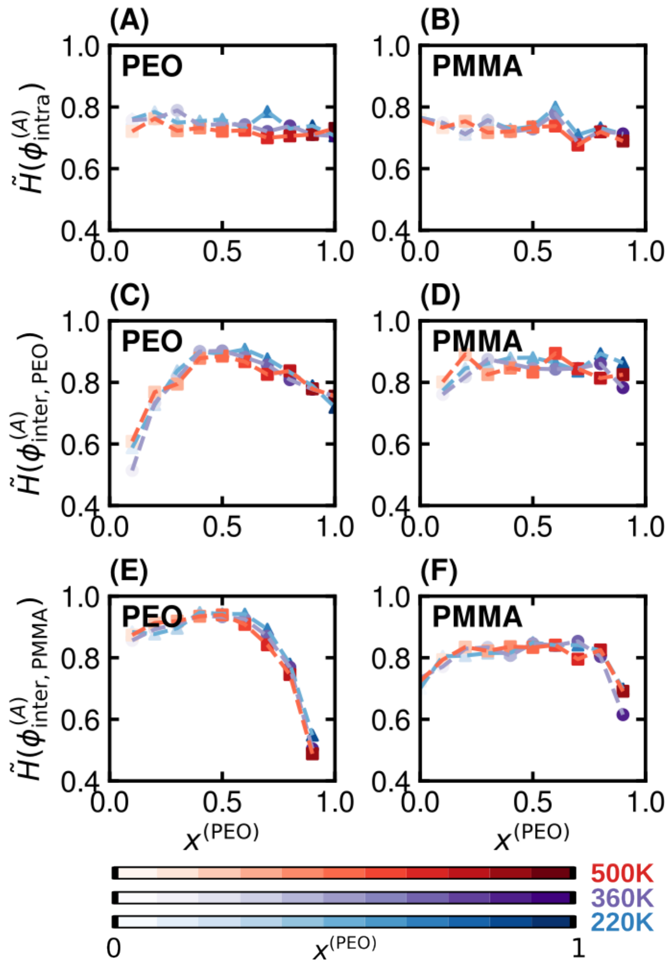

Figure S9: Normalized Shannon entropy of distributions of intra and intermolecular species around backbone carbons of (A,C,E) PEO and (B,D,F) PMMA versus blend composition. The entropies are shown for distributions of (A) intramolecular PEO, (B) intramolecular PMMA, (C,D) intermolecular PMMA, and (E,F) intermolecular PEO within one Kuhn length of the backbone carbons of each species at (red squares) 500 K, (purple circles) 360 K, and (blue triangles) 220 K. Dashed lines provide a guide to the eye.

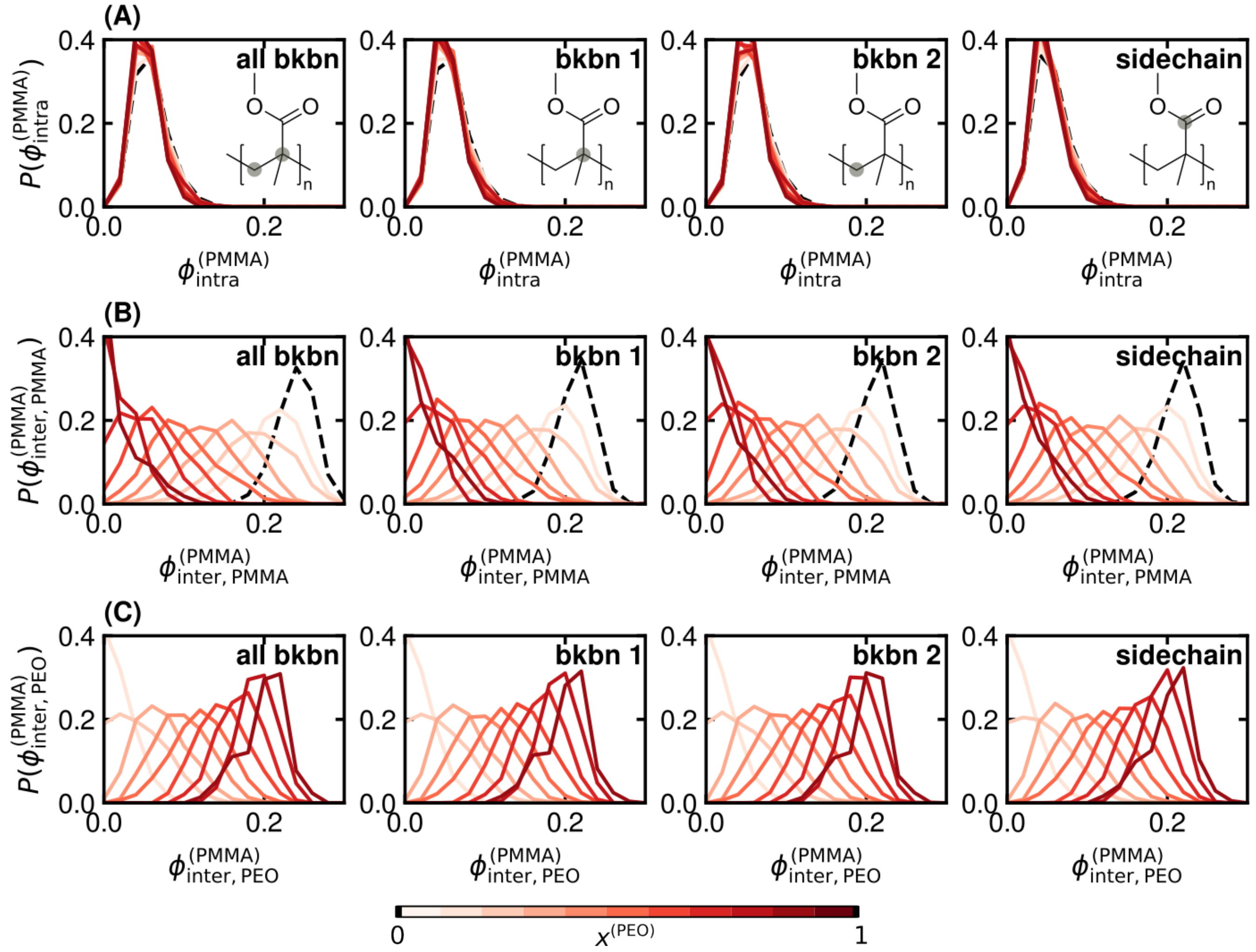

Figure S10: Probability distributions of the volume fraction of (A) intramolecular PMMA, (B) intermolecular PMMA, and (C) intermolecular PEO within a Kuhn length of different carbons on the PMMA chain. Distributions are shown for compositions around all backbone carbons, the quaternary backbone carbon, the secondary backbone carbon, and the ester side chain carbon on PMMA chains. The visual representations of the PMMA atoms around which distributions are characterized shown in (A) apply to all panels within each corresponding column. Results are shown at 500 K. Results for pure systems are shown as black dashed lines.

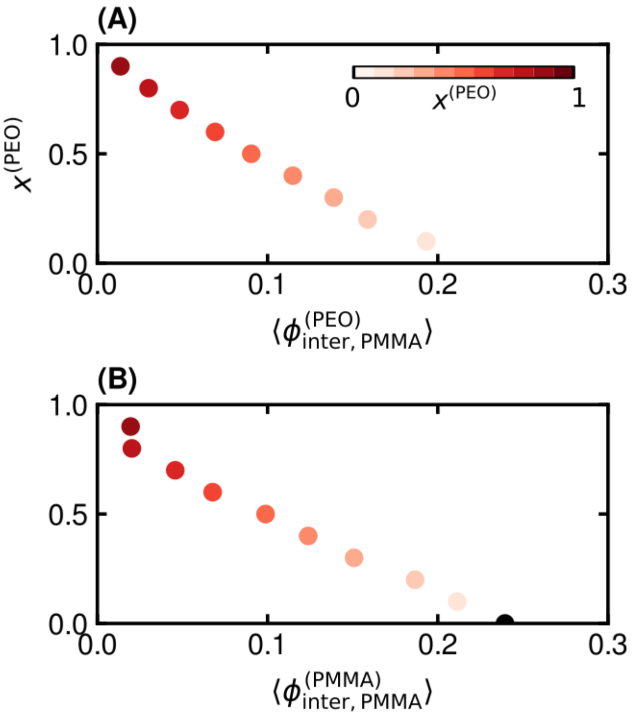

Figure S11: Variation in average intermolecular PMMA volume fractions around (A) PEO and (B) PMMA with blend composition.

S7 Rouse mode analysis

Effective relaxation times and stretching parameters used in the Rouse mode analysis are extracted from normalized Rouse mode ACFs of PEO and PMMA.

Stretching parameters β are fit to the ACFs using three different methods to show that the extracted τ_p^{eff} values are independent of fitting method. In each method, we quantify the adequacy of the fit using the R^2 coefficient of determination. In the first method, we fit the ACFs using fixed values of β that vary from 1 to 0.4. Results are shown in Figure S12. We find that for $\beta = 1$, adequate fits ($R^2 > 0.90$) are achieved readily for pure PEO for $p < 40$ ($N/p > 1.875$) but deviate steadily for lower $x^{(\text{PEO})}$ and smaller N/p. Adequate fits for PMMA are only achieved for $x^{(\text{PEO})} = 0.9$ for $p < 6$ ($N/p > 5.5$) and similarly deviate at low $x^{(\text{PEO})}$ and small N/p. The fixed values of β with the best fits are $\beta = 0.6$ for PEO and $\beta = 0.5$ for PMMA. Representative ACFs from one run per $x^{(\text{PEO})}$ at 500 K and their fits using values of τ_p^{eff} calculated from the linear regression method described in Section 2.2.5 and a fixed value of $\beta = 1$ are shown for PEO and PMMA in Figures S13 and S14, respectively.

Next, we fit the ACFs by allowing the β to vary with p and $x^{(\text{PEO})}$. Results are shown in Figure S15. In this case, R^2 is closest to 1 for all values of p and $x^{(\text{PEO})}$ but β varies significantly across these parameters. Representative ACFs from one run per $x^{(\text{PEO})}$ at 500 K and their fits allowing β to vary with both p and $x^{(\text{PEO})}$ are shown for PEO and PMMA in Figures S16 and S17, respectively.

Finally, we fit the ACFs using L2 regularization weighted to discourage β from deviating from 1. Since the number of data points varies dramatically across p, a λ value of $\alpha/2$ is used, where α is the number of data points available for fitting. Results are shown in Figure S18 and show R^2 values that are worse than the fits for a fully variable β but are better than the fits for $\beta = 1$. They are similar to the values we get from the best-fit fixed β fits. Representative ACFs from one run per $x^{(\text{PEO})}$ at 500 K and their fits using L2 regularization are shown for PEO and PMMA in Figures S19 and S20, respectively.

In each of these three cases, we observe that while β varies widely with fit method, the approximate shapes of the mostly-decayed relaxation times τ_p^{eff} (opaque points) are the same. As a result, any analysis of β would be confounded by the method of the fitting. In Section 3.4, we thus avoid avoid analysis of β and restrict the Rouse analysis to the behavior of τ_p^{eff}, which is not dependent on fitting method. The data shown in Figure 6 result from the unconstrained ACF fitting.

Finally, the collapse of the relaxation times of PMMA Rouse modes onto a master curve is shown in Figure S21 to highlight that collective PMMA dynamics upon blending are shifted by a constant, composition-dependent rescaling factor governed by the distance from T_{g}. The relaxation times in Figure S21 are shifted by a factor of $\exp\left(-\frac{T-T_{\text{g}}(x^{(\text{PEO})}=0.9)-109\text{ K}}{T-T_{\text{g}}(x^{(\text{PEO})})-109\text{ K}}\right)$ to collapse onto $\tau_p^{\text{eff}}(x^{(\text{PEO})} = 0.9)$ where T=500 K. The constant factor of 109 K is used in the shift factor to minimize the mean-squared error of the collapsed relaxation times with $\tau_p^{\text{eff}}(x^{(\text{PEO})} = 0.9)$. For $x^{(\text{PEO})} = 0$ to 0.4, the blend T_{g} values are $\geq$ 393 K causing the shifted relaxation times to deviate because $T - T_{\text{g}}(x^{(\text{PEO})}) - 109\text{ K} < 0$ K. Deviations in collective dynamics are expected close to the blend T_{g}, so the shifted data points for $x^{(\text{PEO})}$ between and including 0 and 0.4 are not shown.

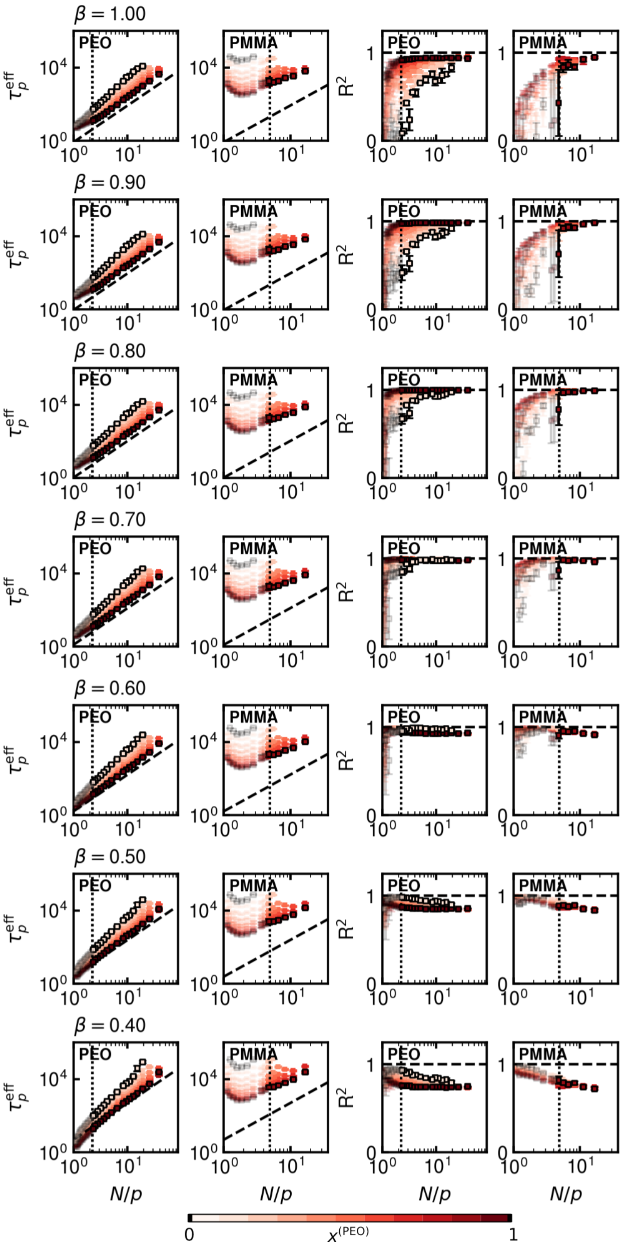

Figure S12: Variations in fit to Rouse mode autocorrelation functions quantified as the R^2 coefficient of determination and resulting relaxation times τ_p^{eff} for PEO and PMMA using a range of fixed β values. The two leftmost columns show the τ_p^{eff} for PEO and PMMA versus sub-chain length N/p while the two rightmost columns show the corresponding R^2 values versus N/p. Data shown is at 500 K. For $p > 8$, symbols for data are only shown for every third value of p for visual clarity. Dashed black lines in the two leftmost columns are a guide to the eye to indicate the expected ideal scaling of $\tau_p \sim p^{-2}$. Results for chains in blends with the most extreme compositions are outlined in black for visual clarity of trends. Transparent markers are used for N/p corresponding to sub-chains equal to or less than an estimated Kuhn length. Dotted black vertical lines mark the N/p corresponding to a sub-chain of Kuhn length size for both PEO and PMMA. Error bars reflect standard errors from three independent systems and are generally smaller than the symbol size.

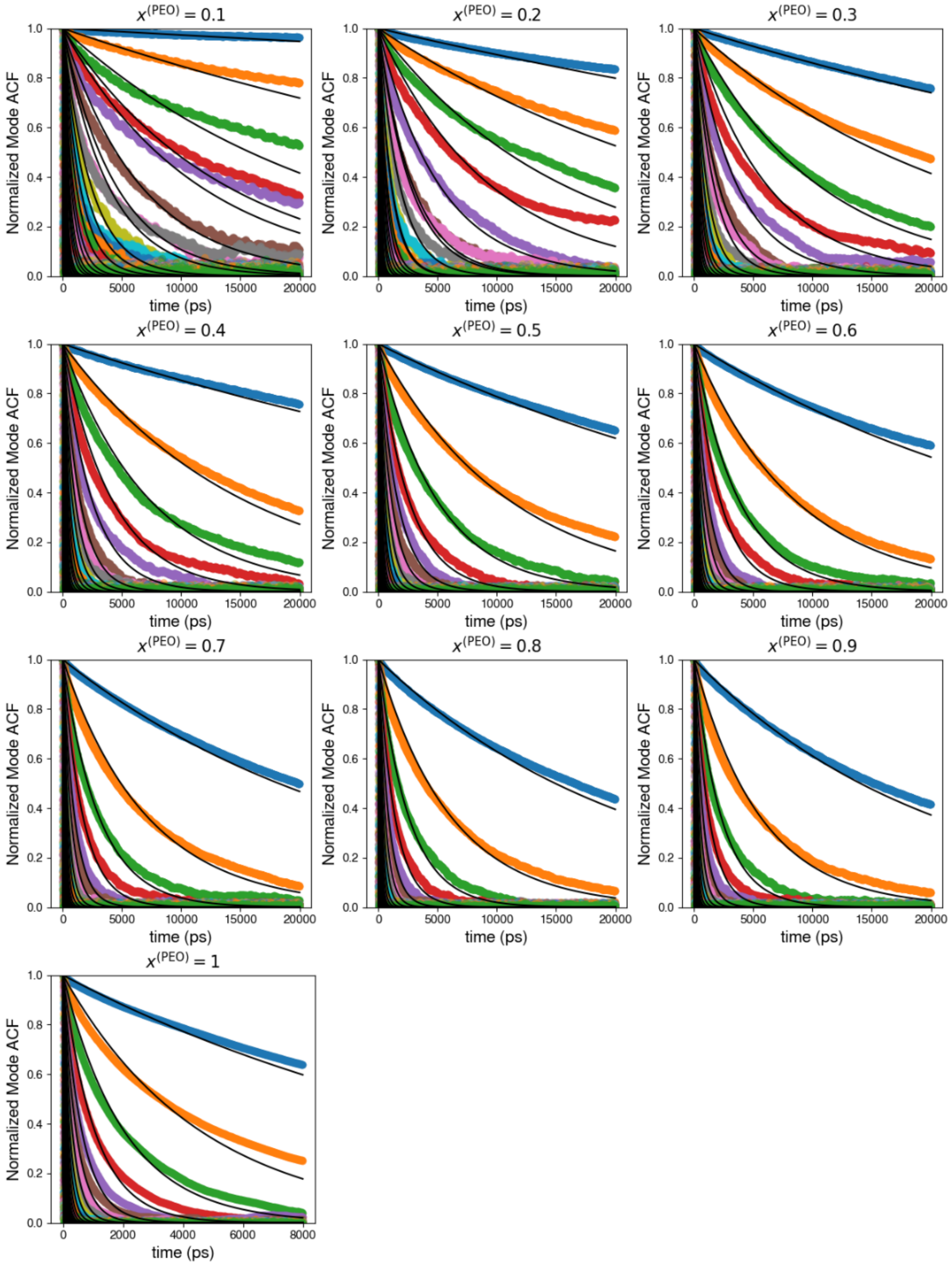

Figure S13: Normalized Rouse mode autocorrelation functions (ACFs) and their fits for PEO chains in the first run of each blend composition. ACFs are fit by fixing $\beta = 1$. Each ACF shown in each panel is for one Rouse mode. The topmost ACF is the ACF for the $p = 0$th Rouse mode, the ACF below it is for the $p = 1$st Rouse mode, and so on. The ACFs of all 74 Rouse modes for PEO are plotted. Black solid lines are fits to each ACF.

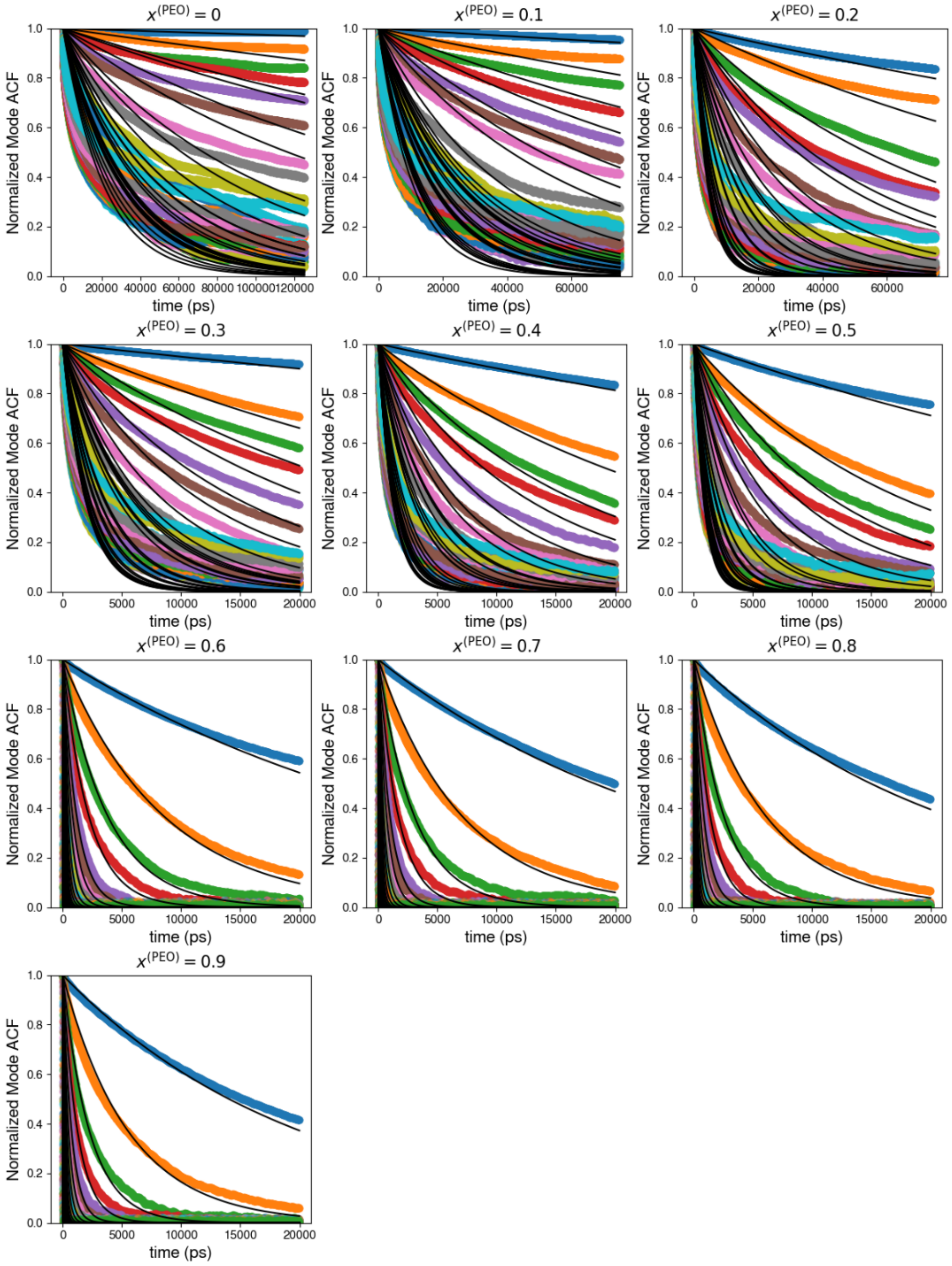

Figure S14: Normalized Rouse mode autocorrelation functions (ACFs) and their fits for PMMA chains in the first run of each blend composition. ACFs are fit by fixing $\beta = 1$. Each ACF shown in each panel is for one Rouse mode. The topmost ACF is the ACF for the $p = 0$th Rouse mode, the ACF below it is for the $p = 1$st Rouse mode, and so on. The ACFs of all 32 Rouse modes for PMMA are plotted. Black solid lines are fits to each ACF.

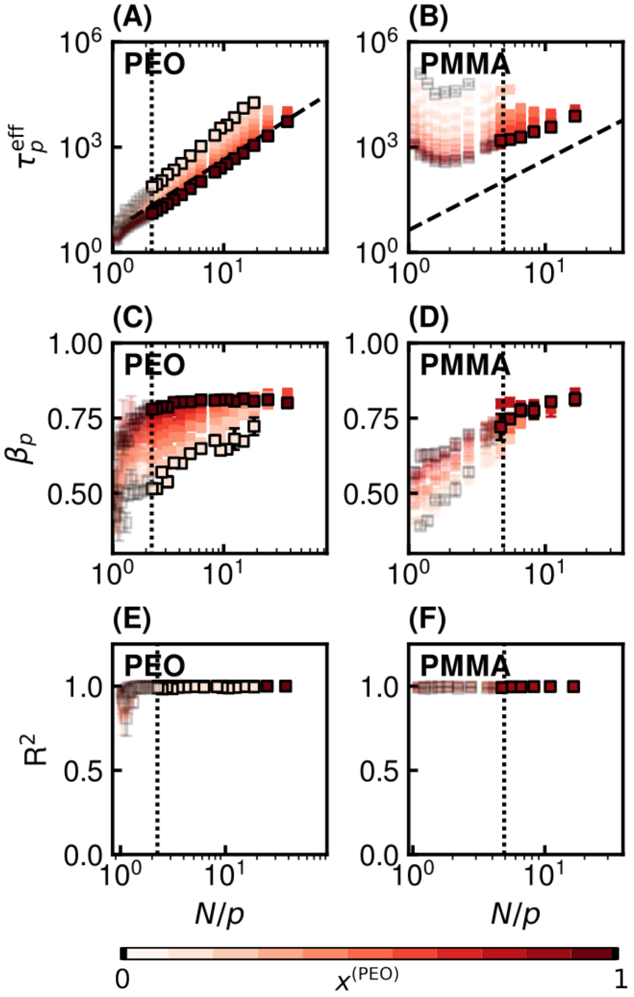

Figure S15: Rouse mode analysis at 500 K for chains in blends of varying composition where β_p is allowed to vary with both p and $x^{(PEO)}$. The effective Rouse relaxation time τ_p^{eff} as a function of sub-chain length N/p for (A) PEO and (B) PMMA. The mode-dependent stretching exponent β_p for (C) PEO and (D) PMMA. R^2 coefficient of determination for the fit of τ_p and β_p to the Rouse mode autocorrelation functions for (E) PEO and (F) PMMA. For $p > 8$, symbols for data are only shown for every third value of p for visual clarity. Transparent data points correspond to Rouse modes that correspond to length scales smaller than the Rouse bead size. Dashed black lines in panels (A) and (B) are a guide to the eye to indicate the expected ideal scaling of $\tau_p \sim p^{-2}$. The position of the line is the same across panels and is set to align with the behavior of neat PEO. Results for chains in blends with the most extreme compositions are outlined in black for visual clarity of trends. Transparent markers are used for N/p corresponding to sub-chains equal to or less than an estimated Kuhn length. Dotted black vertical lines mark the N/p corresponding to a sub-chain of Kuhn length size for both PEO and PMMA. Error bars reflect standard errors from three independent systems and are generally smaller than the symbol size.

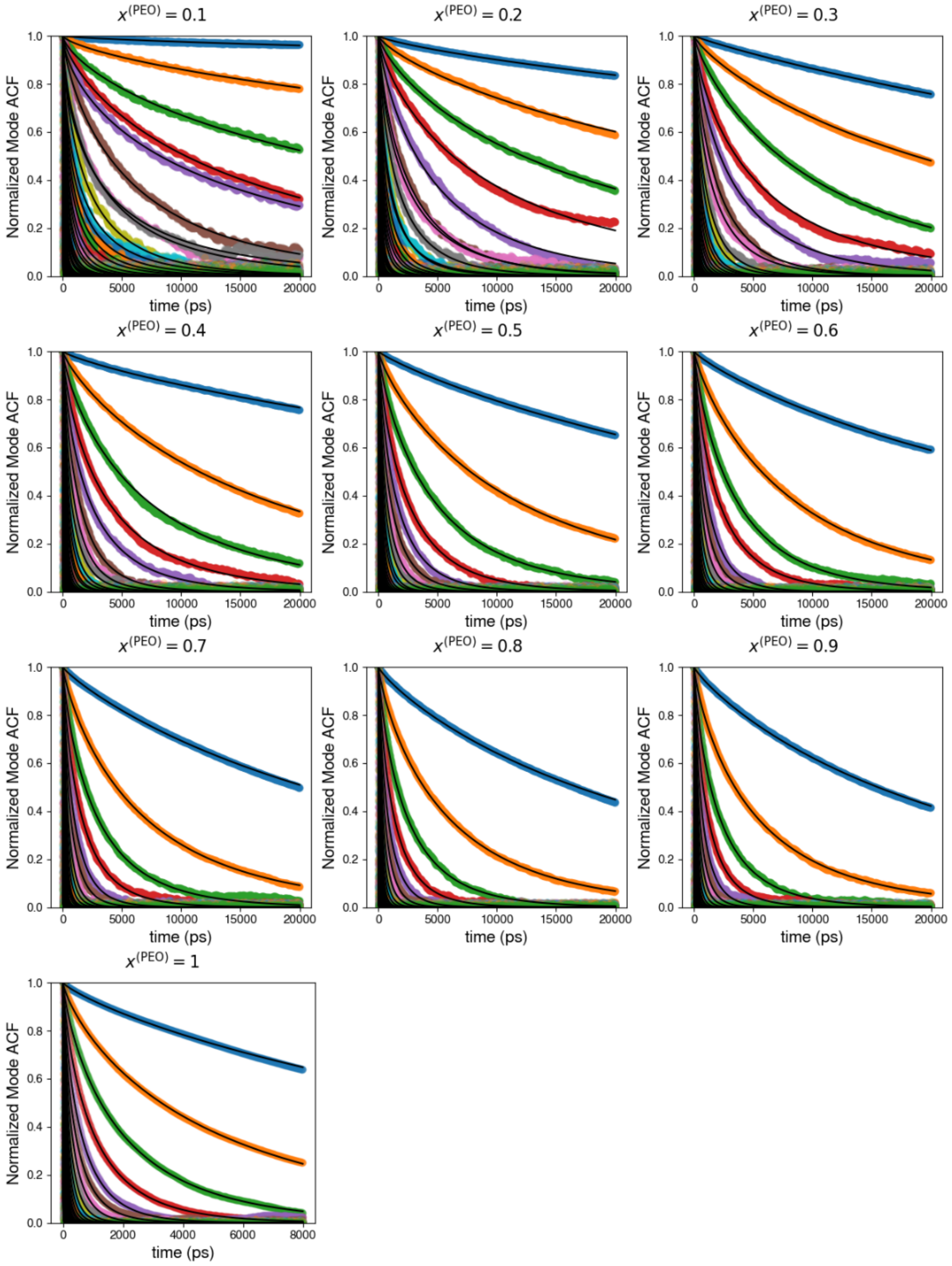

Figure S16: Normalized Rouse mode autocorrelation functions (ACFs) and their fits for PEO chains in the first run of each blend composition. ACFs are fit by allowing β to vary with p and $x^{(PEO)}$. Each ACF shown in each panel is for one Rouse mode. The topmost ACF is the ACF for the $p = 0$th Rouse mode, the ACF below it is for the $p = 1$st Rouse mode, and so on. The ACFs of all 74 Rouse modes for PEO are plotted. Black solid lines are fits to each ACF.

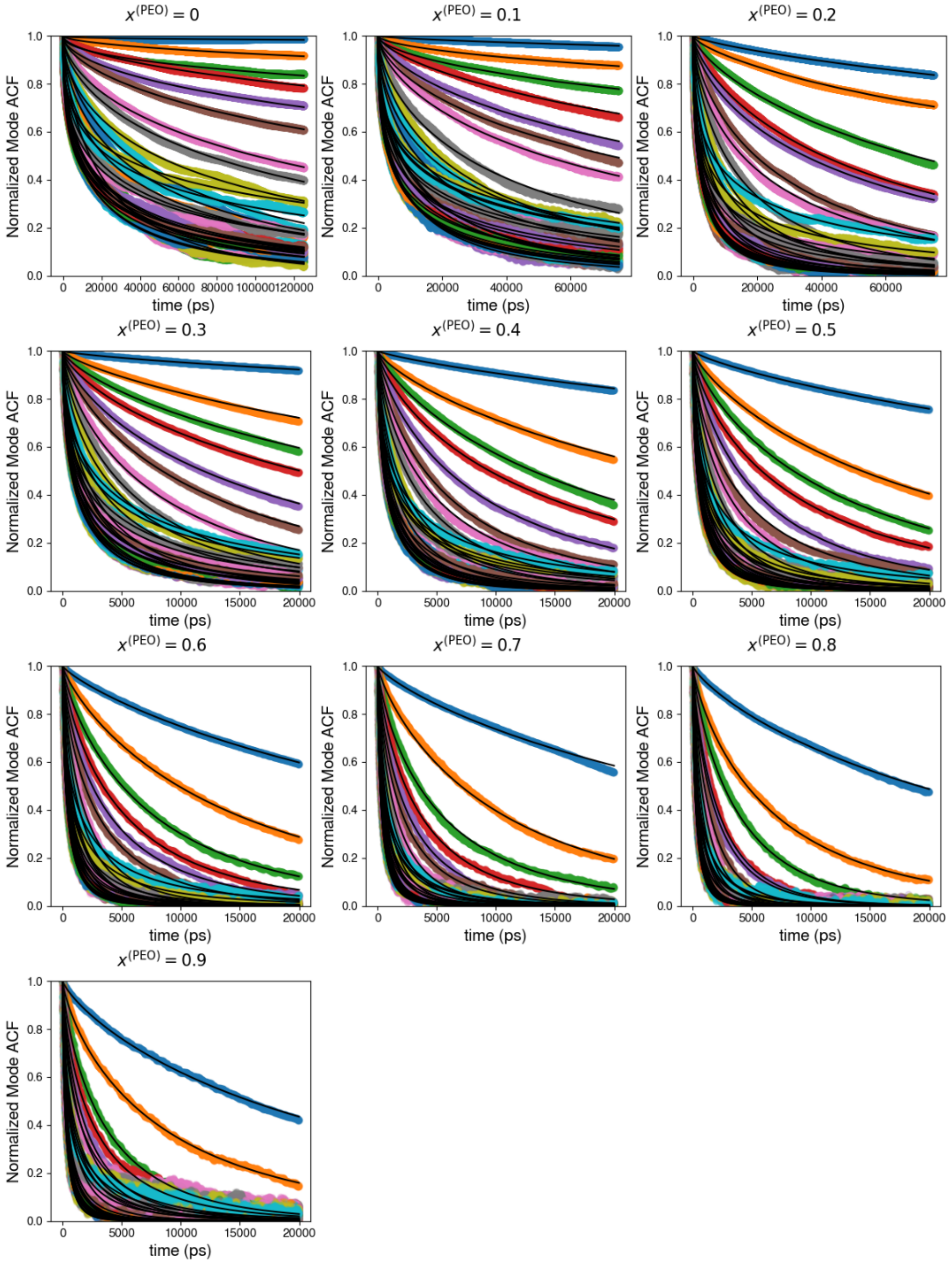

Figure S17: Normalized Rouse mode autocorrelation functions (ACFs) and their fits for PMMA chains in the first run of each blend composition. ACFs are fit by allowing β to vary with p and $x^{(\mathrm{PEO})}$. Each ACF shown in each panel is for one Rouse mode. The topmost ACF is the ACF for the $p = 0$th Rouse mode, the ACF below it is for the $p = 1$st Rouse mode, and so on. The ACFs of all 32 Rouse modes for PMMA are plotted. Black solid lines are fits to each ACF.

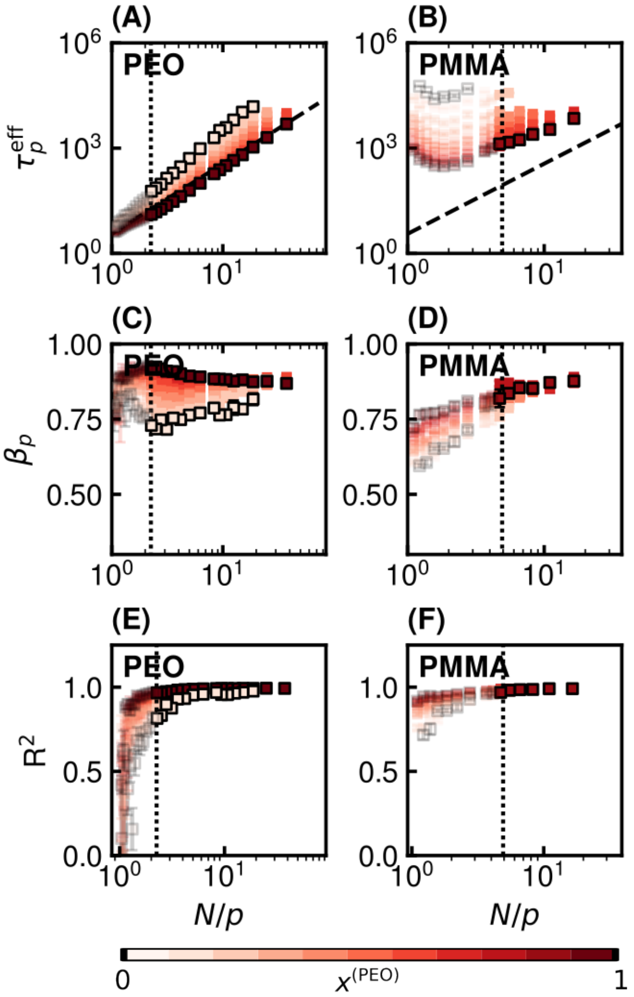

Figure S18: Rouse mode analysis at 500 K for chains in blends of varying composition where β_p is allowed to vary with both p and $x^{(PEO)}$ but is discouraged from deviation from 1 using L2 regularization. The effective Rouse relaxation time τ_p^{eff} as a function of sub-chain length N/p for (A) PEO and (B) PMMA. The mode-dependent stretching exponent β_p for (C) PEO and (D) PMMA. R^2 coefficient of determination for the fit of τ_p and β_p to the Rouse mode autocorrelation functions for (E) PEO and (F) PMMA. For $p > 8$, symbols for data are only shown for every third value of p for visual clarity. Transparent data points correspond to Rouse modes that correspond to length scales smaller than the Rouse bead size. Dashed black lines in panels (A) and (B) are a guide to the eye to indicate the expected ideal scaling of $\tau_p \sim p^{-2}$. The position of the line is the same across panels and is set to align with the behavior of neat PEO. Results for chains in blends with the most extreme compositions are outlined in black for visual clarity of trends. Transparent markers are used for N/p corresponding to sub-chains equal to or less than an estimated Kuhn length. Dotted black vertical lines mark the N/p corresponding to a sub-chain of Kuhn length size for both PEO and PMMA. Error bars reflect standard errors from three independent systems and are generally smaller than the symbol size.

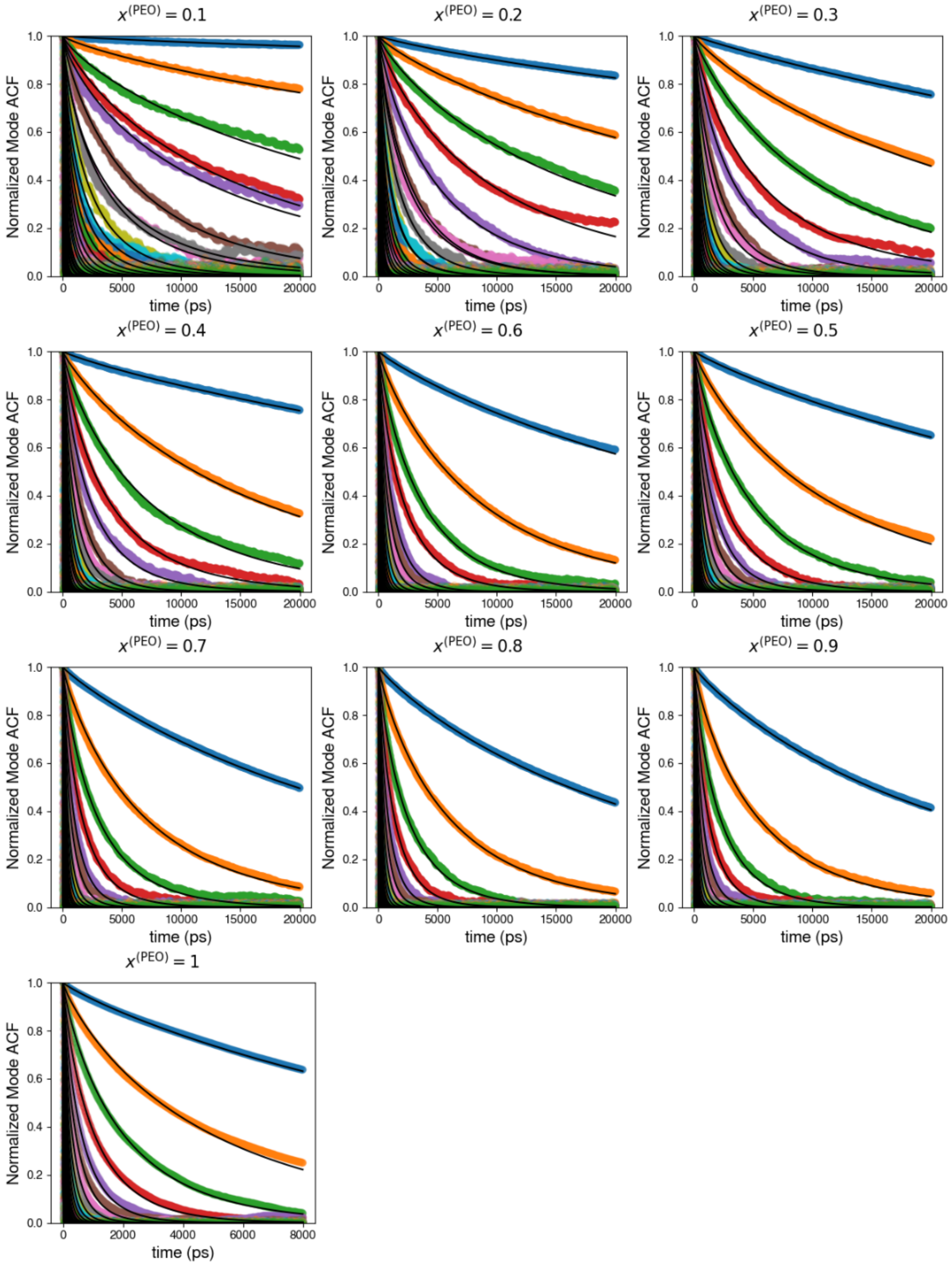

Figure S19: Normalized Rouse mode autocorrelation functions (ACFs) and their fits for PEO chains in the first run of each blend composition. ACFs are fit using L2 regularization. Each ACF shown in each panel is for one Rouse mode. The topmost ACF is the ACF for the $p = 0$th Rouse mode, the ACF below it is for the $p = 1$st Rouse mode, and so on. The ACFs of all 74 Rouse modes for PEO are plotted. Black solid lines are fits to each ACF.

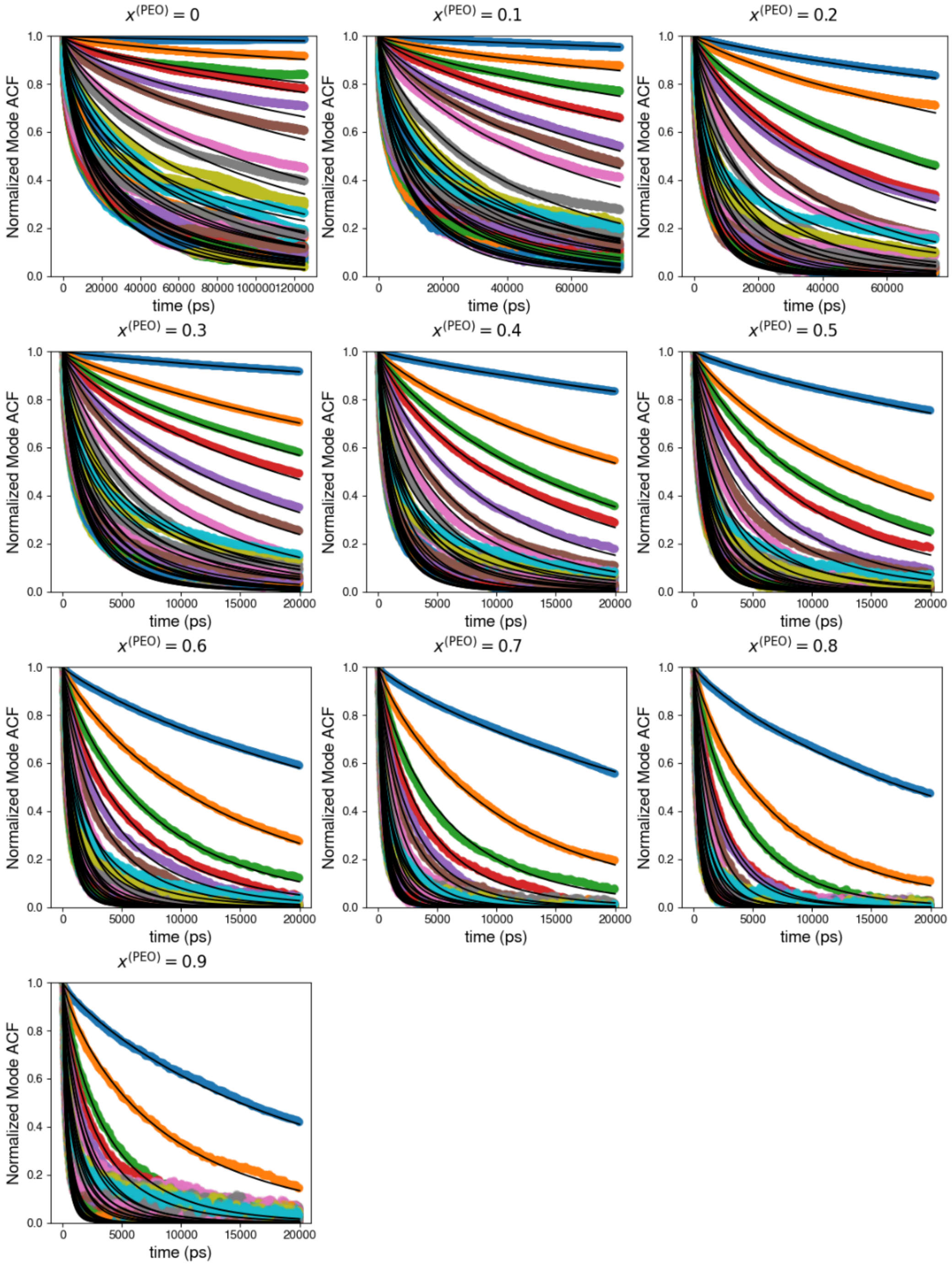

Figure S20: Normalized Rouse mode autocorrelation functions (ACFs) and their fits for PMMA chains in the first run of each blend composition. ACFs are fit using L2 regularization. Each ACF shown in each panel is for one Rouse mode. The topmost ACF is the ACF for the $p = 0$th Rouse mode, the ACF below it is for the $p = 1$st Rouse mode, and so on. The ACFs of all 32 Rouse modes for PMMA are plotted. Black solid lines are fits to each ACF.

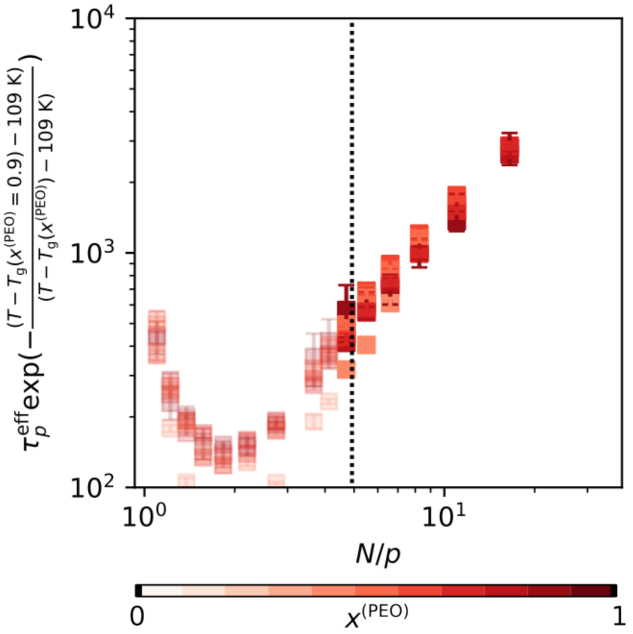

Figure S21: Shifted relaxation times of PMMA Rouse modes. Results are shown only for $x^{(\mathrm{PEO})} \geq 0.5$, as the shifted relaxation times for $x^{(\mathrm{PEO})} < 0.5$ deviate from the master curve. For $p > 8$, symbols for data are only shown for every third value of p for visual clarity. Transparent data points correspond to Rouse modes that correspond to length scales smaller than the Rouse bead size. The dotted black vertical line marks the N/p corresponding to a sub-chain of Kuhn length size of PMMA. Error bars reflect standard errors from three independent systems and are generally smaller than the symbol size.
